# PINN for stiff moving-boundary PDE to predict the locking point in superheated steam drying

**DOI:** 10.1038/s41598-026-59320-1

**Published:** 2026-06-23

**Authors:** Narjes Malekjani, Andreas Bück, Evangelos Tsotsas, Abdolreza Kharaghani

**Affiliations:** Thermal Process Engineering, Otto von Guericke University Magdeburg, Universitätsplatz 2, 39106 Magdeburg, Germany

**Keywords:** Advection–diffusion, Residual scaling, Physics-informed neural networks, Stiffness stabilization, Engineering, Mathematics and computing, Physics

## Abstract

Physics-informed neural networks (PINNs) often struggle to solve stiff partial differential equations (PDEs) with moving boundaries, such as convection-dominated droplet drying with shrinking domains and related Stefan-type phase-change problems. This study investigates a physics-guided PINN formulation for nanosuspension droplet drying in superheated steam, where an accurate prediction of the locking point is important, as it marks the onset of structure formation and significantly influences particle morphology and final powder characteristics. The formulation retains the standard PINN backbone and combines two problem-specific modifications: a logarithmic state-variable transformation to compress the solution dynamic range and improve the representation of steep near-surface gradients, and a Péclet number-based scaling of the surface residual to better balance the boundary and PDE training signals across the stiffness regimes. Compared with a numerically verified Crank–Nicolson (CN) reference solution, the proposed formulation showed the greatest advantage in the high-Péclet regime, where the boundary layer steepening and training difficulty were most pronounced. In the stiffest case considered, it substantially reduced the discrepancy from the numerically verified CN reference solution compared with both the baseline PINN and the unscaled log-transformed formulation, while also improving the locking-time prediction. These results show that a physics-guided state transformation, combined with residual scaling, can improve the robustness of PINNs for this class of stiff, moving-boundary drying problems.

**Supplementary Information:**

The online version contains supplementary material available at 10.1038/s41598-026-59320-1.

## Introduction

Spray drying of nanosuspensions is a crucial process for producing functional powders in the food, pharmaceutical, and materials engineering industries^1^. The resulting particle morphology may be a dense sphere, a hollow shell, or a porous agglomerate, which is directly linked to the internal solute concentration profiles and dynamics of structure formation during drying^2^. Superheated steam drying has several advantages over conventional hot air drying, including higher energy efficiency, reduced oxidative degradation, and modified drying kinetics^2,3^.

**Fig. 1:**
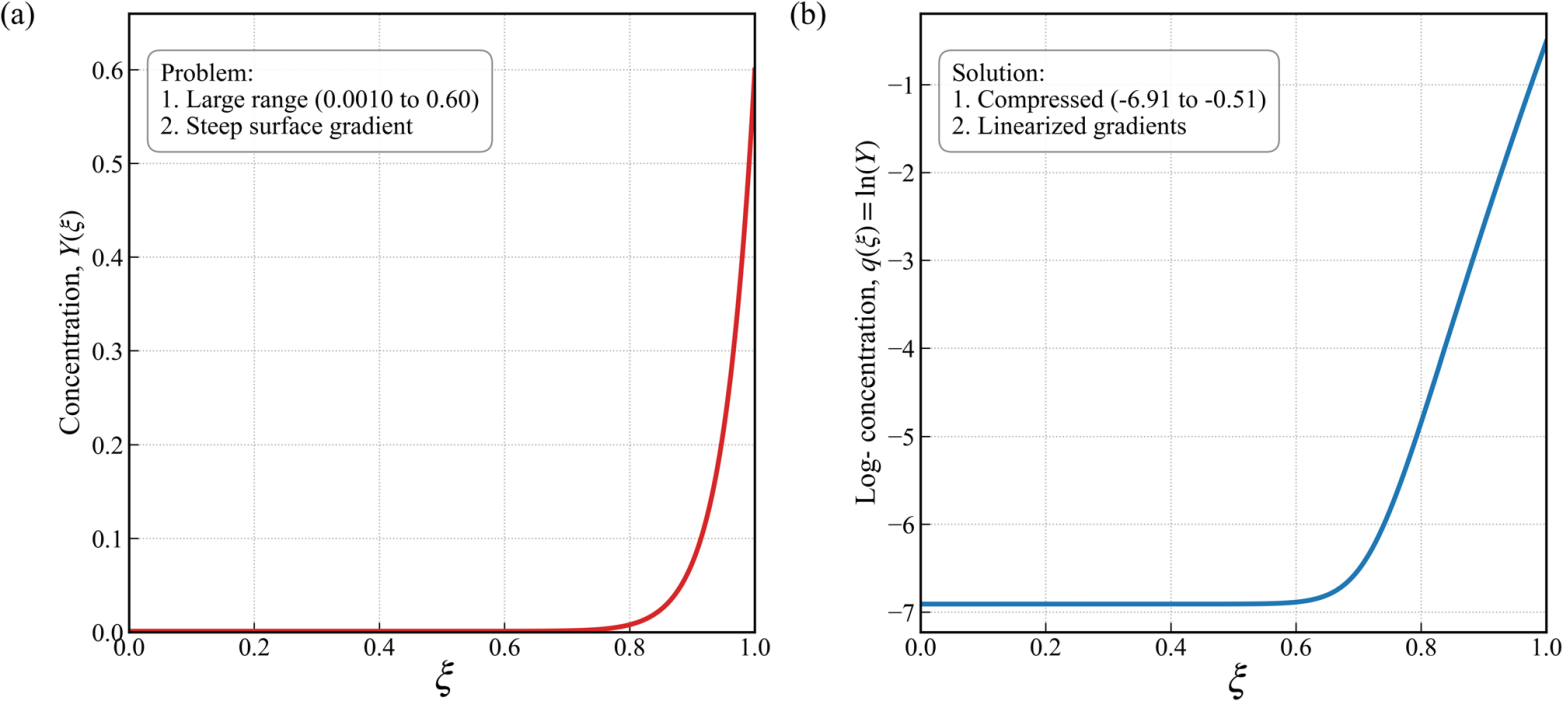
Effect of logarithmic transformation on stiffness in the concentration profile: (**a**) Original space (*Y*-space), (**b**) Transformed space (*q* = *ln(Y)*).

Recent studies on nanosuspension droplets in superheated steam have demonstrated that solvent evaporation, nanoparticle diffusion, and crust formation (“locking”) can be modeled using a nonlinear advection–diffusion model in spherical geometry with a time-dependent boundary^2^. This model successfully predicts the impact of the superheating level and convective conditions on the shell thickness, porosity, and final particle structure. In these regimes, the solute mass fraction profile exhibits exponential boundary-layer steepening near the droplet surface (Fig. 1a)^2^. Therefore, an accurate prediction of the locking point is important because it marks the onset of crust formation and affects the final particle morphology and powder properties. In the present problem, stiffness arises because drying produces a sharp, localized near-surface concentration gradient, whereas the droplet interior remains comparatively smooth and slowly varying. This large spatial contrast makes it difficult to approximate the solution uniformly.

Scientific machine learning methods have recently attracted increasing attention in several fields of science and engineering. Physics-informed neural networks (PINNs) are a scientific machine-learning approach for solving forward and inverse problems governed by partial differential equations (PDEs). They embed the governing equations, initial conditions (ICs), and boundary conditions (BCs) directly into the training process^4^. Once trained, a PINN can serve as a mesh-free surrogate model capable of fast evaluations within trained operating conditions. These advantages have motivated the widespread application of PINNs in fluid mechanics, solid mechanics, process engineering, and other fields ^5–8^.

Despite these benefits, many recent studies have demonstrated that standard PINNs struggle with time-dependent transport phenomena, moving-boundary problems, and stiff PDEs because of issues such as loss imbalance, spectral bias, and causality violations, often resulting in collapse to trivial solutions^9–11^. This failure was explicitly demonstrated in single-droplet drying, where standard PINNs failed to propagate the internal concentration gradients over time^12^. This behavior is consistent with the tendency of PINNs to learn low-frequency components before high-frequency or highly localized features^13^. Wang et al.^14^ further analyzed PINN training failures from an NTK perspective. They showed that imbalanced convergence rates among the IC, BC, and PDE losses can prevent PINNs from accurately learning the full solution field.

Several approaches have been proposed to address these limitations. The first is the adaptive sampling method. Wu et al.^15^ introduced a residual-based adaptive distribution and residual-based adaptive refinement with distribution methods that dynamically redistribute collocation points according to the PDE residual during training. Daw et al.^9^ proposed a retain-resample-release (R3) strategy that concentrates the training effort in regions with high PDE residuals. Guo et al.^16^ proposed a temporal-causality-based adaptive sampling method for time-dependent PDEs that improves PINN performance by selecting collocation points consistent with the causal evolution of the solution in time. Although these methods are effective, they add computational complexity and require residual monitoring. The second line of work modifies the solution representation, network decomposition, or training strategy. For example, finite-basis PINNs^17^ divide the computational domain into smaller overlapping regions and use local neural networks in each region. In contrast, parameter-asymptotic and parameter-driven PINN strategies^18,19^ start from easier problems with larger perturbation parameters and gradually move toward more difficult singularly perturbed cases with sharp boundaries or interior layers.

The third strategy in the PINN literature involves directly addressing gradient–flow pathologies. Wang, Teng, and Perdikaris^20^ demonstrated that PINN training can suffer from numerical stiffness when the back-propagated gradients associated with different loss terms, such as BC, IC, and PDE residual losses, are highly imbalanced. They proposed a learning-rate annealing strategy that employs gradient statistics during training to balance the contributions of different loss components adaptively. However, such adaptive balancing introduces computational overhead and additional algorithmic choices.

Other strategies have also aimed to improve PINN performance through curriculum regularization, richer coordinate embeddings, domain decomposition, or operator learning. Krishnapriyan et al.^10^ analyzed possible PINN failure modes and demonstrated that such failures can arise from ill-conditioning and optimization difficulties associated with soft PDE constraints, rather than from the inability of the neural network to represent the solution. They proposed curriculum regularization, in which the PDE regularization starts from a simpler form and gradually becomes more complex during training, as well as a sequence-to-sequence formulation that avoids learning the full spatiotemporal solution at once. Wang, et al.^11^ introduced spatiotemporal and multiscale random Fourier feature embeddings to improve PINN performance for high-frequency or multiscale solution structures. Decomposition-based methods, such as XPINNs, have also been proposed by other researchers. These methods use space–time domain decomposition with separate neural networks in subdomains, thereby increasing the representation capacity and enabling parallelization^21^. Operator-learning approaches, such as physics-informed DeepONets, are novel methods that aim to learn solution operators for parametric PDEs while imposing physical laws through soft penalty constraints during training^22^.

The fourth and most recent approach for singularly perturbed or convection-dominated PDEs is the input transformation. Guan and Elman^23^ studied singularly perturbed convection–diffusion problems, which are characterized by steep boundary layers that are difficult to resolve, and examined the use of PINNs with input transformations to enhance accuracy. They also analyzed the behavior of input transformations using neural tangent kernel arguments. However, an important question remains regarding the current superheated steam drying benchmark. Although input transformations can improve the representation of sharp spatial features, they primarily act through the parameterization of the input domain and do not directly transform the dependent variable itself. In the present drying problem, the large dynamic range and rapid near-surface growth of the dependent variable remain challenging for the network to learn. Related classical numerical studies have also highlighted the difficulty of convection-dominated, stiff, and moving-boundary problems. This issue is particularly relevant for convection-dominated PDEs, where specialized bound-preserving finite element formulations have been developed to ensure that discrete solutions respect physical bounds^24^. Recent classical numerical studies have also addressed challenging nonlinear, fractional, oscillatory, higher-order, and stiff differential models using specialized discretization strategies, including the 2D time-fractional Burgers equation^25^, Kuramoto–Sivashinsky equation^26^, fourth-order reaction–diffusion equations^27^, and stiff nonlinear ordinary differential equations solved using hybrid block methods^28^. Recent classical studies have also examined nonlinear phase change and moving-boundary problems with temperature-dependent or spatially varying thermophysical properties, including one- and two-phase formulations in functionally graded media and sublimation models with variable permeability, internal heat generation, radiation, and convective boundary effects^29–32.^. These studies demonstrate the importance of problem-specific formulations for difficult transport and moving-boundary problems. However, they focus on classical solver development and do not address PINN-specific training pathologies, such as loss imbalance, spectral bias, and boundary-residual dominance.

In summary, existing PINN remedies mainly act through sampling, adaptive loss balancing, architecture, domain decomposition, operator learning, or coordinate parameterization, whereas classical numerical studies rely on specialized discretization strategies. Therefore, the key question in this study is whether such a formulation-level modification can improve the robustness of the PINN for the present stiff moving-boundary drying PDE. In this study, we address this issue using a standard PINN backbone augmented with two physics-guided components: logarithmic state-variable transformation and boundary-residual scaling. For brevity, the log-transformed formulation is referred to as LT-PINN, and the log-transformed formulation with boundary-residual scaling is referred to as scaled LT-PINN. These labels denote formulation variants built on the same standard PINN backbone rather than different neural network architectures. The objective was to determine whether these formulation-level modifications improve the robustness and accuracy relative to the same PINN backbone applied directly to the physical variable.

The proposed formulation consists of two main components.

1. Logarithmic state-variable transformation (LT-PINN): Unlike input coordinate transformations, such as those in^23^ or domain decomposition methods, we apply a state-variable transformation *q* = *ln Y*, where *Y* is the solute mass fraction. This transformation directly affects the dependent variable, and in the present drying problem, (a) compresses the dynamic range of the output, converting exponential growth into an approximately linear profile, and (b) transforms the numerically stiff Robin BC for solute mass fraction (Y) into a simpler BC for the transformed *q*. This modifies the PDE form and BC learned by the network in ways that cannot be achieved through input preprocessing or adaptive sampling alone.

2. Physics-informed BC residual scaling (scaled LT-PINN): Unlike adaptive approaches that correct imbalances iteratively (e.g., Wang et al.^20^), we do not propose a new general adaptive balancing algorithm in this study. Instead, we propose a direct, physics-informed residual-scaling strategy for the drying problem that adjusts the contribution of the surface BC residual based on domain-specific physical knowledge. For problems in which a clear stiffness parameter exists (such as the Péclet number, *Pe*, in single-droplet drying, which represents the ratio of evaporative transport to internal solute diffusion), this method provides a simple alternative that helps improve the balance between the PDE and boundary contributions while remaining physically interpretable.

To the best of our knowledge, this specific combination of a logarithmic state-variable transformation and physics-informed BC residual scaling has not been systematically examined for the current superheated steam single-droplet drying benchmark in a PINN setting. Thus, the contribution of this study is a benchmarked formulation-level strategy aimed at addressing a clear gap in the PINN treatment of stiff moving-boundary drying PDEs.

The remainder of this paper is structured as follows: The "Spatially-resolved modeling of single-droplet drying in superheated steam" section presents the the governing equations, IC and BCs, the transformation from a moving to a fixed computational domain, and the derivation of the transformed formulation. The “Implementation of PINNs ” section introduces the compared PINN formulations, the details of the shared network backbone, loss terms, and BC residual scaling. The "Results and discussion" section reports the results of the ablation study comparing these formulations in terms of concentration profiles and the locking point in single-droplet superheated steam drying. The "Future perspectives" section outlines future perspectives, and the “Conclusion” section summarizes the main conclusions.

## Spatially-resolved modeling of single-droplet drying in superheated steam

The drying of a nanosuspension or colloidal droplet in superheated steam involves the simultaneous transport of water vapor out of the droplet and the diffusion of suspended nanoparticles within the liquid phase. As evaporation progresses, the droplet radius decreases, and the nanoparticle concentration increases until the locking point is reached, at which point the particles form a rigid structure near the surface^2^.

The governing equations in this section follow the standard shrinking droplet diffusion framework of Brenn^33^, whereas the superheated steam shrinkage relations and locking-point interpretation follow Bück^2^. The equations obtained later by front-fixing, nondimensionalization, and time reparameterization are derived from these base equations. The derivation steps are summarized here and detailed in Appendices A and B.

The formulations used in this study were restricted to the pre-locking stage and should be interpreted as a single-phase, pre-crust model. In this setting, the moving boundary addressed in the governing equation is the shrinking outer droplet radius, whereas the locking point is treated as a threshold that marks the onset of structure formation at the surface. The model does not explicitly track the post-locking internal phase interface, shell growth front, or multiphase region. Accordingly, once locking is reached, the present PDE is no longer expected to remain valid, and a more complete shell-controlled or multiphase moving-boundary formulation is required in the subsequent drying stage.

Assuming a spherical geometry, appropriate for an isolated shrinking droplet, and radial symmetry of the solid distribution within the droplet, the diffusion equation for the substance *i* in the droplet, expressed in terms of mass fraction ${Y}_{i}$, is given by^33^:

1 $$\frac{\partial {Y}_{i}}{\partial t}=\frac{D}{{r}^{2}}\frac{\partial }{\partial r}\left({r}^{2}\frac{\partial {Y}_{i}}{\partial r}\right)$$

where D is the binary diffusion coefficient of the particulates in the liquid. Equation (1) applies within the radial domain $r\in \left[0,a\left(t\right)\right]$, where $a\left(t\right)$ denotes the shrinking time-dependent droplet radius. The binary diffusion coefficient for the diffusion of nanoparticles in this study was evaluated using the Stokes–Einstein relationship:

2 $$D = \frac{{k}_{B}{T}_{d}}{6\pi {\eta}_{l}{r}_{p}}$$

where ${k}_{B}$ is the Boltzmann constant, ${T}_{d}$ is the boiling temperature of the liquid, ${\eta}_{l}$ is the dynamic viscosity of the nanosuspension, and ${r}_{p}$ is the nanoparticle radius.

Under quasi-static conditions and neglecting the thermal radiation effects on evaporation, droplet shrinkage is described by the modified d^2^-t law reported by Bück^2^:

3 $${d}^{2}\left(t\right) ={d}_{0}^{2} -\kappa t$$

where ${d}_{0}$ and *d* are the initial droplet diameter and diameter at time t, respectively. The rate of shrinkage due to evaporation is denoted by $\kappa$:

4 $$\upkappa =8\left(\frac{Nu {\lambda}_{vap}\Delta T}{\Delta {h}_{v}{\rho}_{l}}\right)$$

where $Nu$ denotes the Nusselt number, ${\lambda}_{vap}$ denotes the thermal conductivity of the superheated vapor, $\Delta T$ denotes the superheating temperature, $\Delta {h}_{v}$ denotes the specific evaporation enthalpy, and ${\rho}_{l}$ denotes the mass density of liquid water. The temporal evolution of $a\left(t\right) = d\left(t\right)/2$ is governed by

5 $$\frac{d{a}^{2}}{dt} = \frac{-\kappa }{4}$$

The BC at the droplet surface enforces a balance between the internal transport and interfacial flux and is formulated as follows:

6 $$-D\frac{\partial {Y}_{i}}{\partial r}-\frac{da}{dt}{Y}_{i}=\frac{{\dot{m}}_{i}}{A{\rho}_{l}}$$

where *A* is the droplet surface area and ${\dot{m}}_{i}$ is the mass transfer rate of substance *i* across the droplet surface into the surrounding gas phase. The first term on the left-hand side of Eq. (6) denotes diffusion within the droplet, and the second term accounts for the convective contribution owing to the moving boundary. The right-hand side denotes the mass flux leaving the droplet surface, which is zero for non-evaporating solid. At the center of the droplet, the regularity condition must be satisfied; that is, the radial gradient of the mass fraction profile of the species is zero for all times *t*:

7 $$\frac{\partial {Y}_{i}}{\partial r} = 0$$

The IC is defined as follows:

8 $${Y}_{i}\left(t=0,r\right)={Y}_{i0}\left(r\right)$$

The locking point is reached when the surface solid mass fraction of the droplet reaches a critical value (0.7757, which corresponds to a solid volume fraction of 0.6).

Before introducing the coordinate transformations, it is useful to highlight the main sources of numerical difficulty in this model. Droplet shrinkage induces a time-dependent domain and a moving surface boundary, which couples the interfacial condition to interior transport. Moreover, as drying proceeds, the solute field becomes increasingly convection-dominated near the surface, resulting in a progressively thinner boundary layer with steep concentration profile gradients. The relative strength of this evaporation-driven convective transport compared to diffusion can be characterized by *Pe*. In the diffusion-dominated limit (*Pe → 0*), the concentration profiles remain comparatively smooth, whereas in the convection-dominated limit (*Pe → ∞*), strong surface enrichment and sharp near-surface gradients develop. Accordingly, increasing *Pe* is associated with increasing stiffness in the moving-boundary drying problem. These features motivate the transformations introduced in the following sections: the coordinate and time transformations map the moving-domain problem to a more convenient fixed-domain form, whereas the logarithmic state-variable transformation addresses the large dynamic range and steep near-surface growth of the solution. The benchmark cases considered in this study can therefore be interpreted along a low-to-high *Pe* spectrum, ranging from relatively smooth diffusion-dominated transport to increasingly sharp near-surface gradients in more convection-dominated regimes.

### Radius transformation to handle moving BC

The first transformation applied in this study was the conversion of the time-dependent spatial domain (the volume of the spherical droplet) to a fixed domain to avoid the difficulties associated with a moving BC. Following Brenn^33^, the transformation $\xi =r/a\left(t\right)$ was applied. Accordingly, Eq. (1) was converted into a nonlinear advection–diffusion PDE using the front-fixing method (details are provided in Appendix A):

9 $${a}^{2}\frac{\partial {Y}_{i}}{\partial t}=\frac{d{a}^{2}}{dt}\frac{\xi }{2}\frac{\partial {Y}_{i}}{\partial \xi }+\frac{D}{{\xi }^{2}}\frac{\partial }{\partial \xi }\left({\xi }^{2}\frac{\partial {Y}_{i}}{\partial \xi }\right)$$

This transformation converts the moving spatial domain $r\in \left[0,a\left(t\right)\right]$ into a fixed domain $\xi \in \left[\mathrm{0,1}\right]$. The first term on the right-hand side of Eq. (9) is an advection-like drift term introduced by the front-fixing transformation, and it accounts for the effect of droplet shrinkage in fixed computational coordinates. The surface BC, center regularity condition, and IC transform accordingly. Equation (6) becomes:

10 $$-D\frac{\partial {Y}_{i}}{\partial \xi } + \frac{\kappa }{8}{Y}_{i} = \frac{\dot{{m}_{i}}}{4\pi a{\rho}_{l}}$$

The regularity condition at $\xi = 0$ is:

11 $$\frac{\partial {Y}_{i}}{\partial \xi } = 0$$

and the IC becomes:

12 $${Y}_{i}\left(t=0,\xi \right)={Y}_{i0}\left(\xi \right)$$

The whole problem can then be nondimensionalized with respect to the droplet lifetime.

*t*_l_, defined in the adopted drying-model formulation^33^, as:

13 $${t}_{l} = \frac{-4{a}_{0}^{2}}{\kappa }\left[{\left(\frac{{Y}_{0}}{\left(1-{Y}_{0}\right){\rho}_{s}/{\rho}_{l}+ {Y}_{0}}\right)}^{ 2/3}- 1\right]$$

where ${a}_{0}$ is the initial droplet radius, ${Y}_{0}$ is the initial mass fraction of the solute in the droplet, and ${\rho}_{s}$ is the solid density. Equation (13) assumes that at the end of the drying process, the particle density is equal to the compact solid density ${\rho}_{s}$. Therefore, Eq. (9) can be nondimensionalized as follows:

14 $${\overline{a} }^{2}\frac{\partial {Y}_{i}}{\partial \tau }=\alpha \frac{\xi }{2}\frac{\partial {Y}_{i}}{\partial \xi }+\frac{G}{{\xi }^{2}}\frac{\partial }{\partial \xi }\left({\xi }^{2}\frac{\partial {Y}_{i}}{\partial \xi }\right)$$

where:

$$\overline{a } = \frac{a}{{a}_{0}}, \tau = \frac{t}{{t}_{l}}, \alpha = \frac{-\kappa {t}_{l}}{{4a}_{0}^{2}}, G =\frac{{t}_{l} D}{{a}_{0}^{2}}$$

The BC (Eq. (10)) can be rewritten as follows:

15 $$-\frac{\partial {Y}_{i}}{\partial \xi } + \frac{\kappa }{8D}{Y}_{i} = \frac{\dot{{m}_{i}}}{4\pi aD{\rho}_{l}}$$

and the IC becomes:

16 $${Y}_{i}\left(\tau =0,\xi \right)={Y}_{i0}\left(\xi \right)$$

If the droplet evaporates according to the d^2^-t law, its nondimensional form can be expressed as follows:

17 $$\frac{{a}^{2}\left(t\right)}{{a}_{0}^{2}} = 1+\alpha \tau$$

Defining

18 $$Pe = \frac{\kappa }{8D} = \frac{-\alpha }{2G}$$

Equation (14) becomes:

19 $$\frac{\partial {Y}_{i}}{\partial \tau } = \frac{G}{1+ \alpha \tau }\left[\frac{{\partial }^{2}{Y}_{i}}{\partial {\xi }^{2}}+\left(\frac{2}{\xi }-Pe \xi \right)\frac{\partial {Y}_{i}}{\partial \xi }\right]$$

Because no evaporation occurs for the solute (species 2), $\dot{m} = 0$ and Eq. (15) becomes:

20 $$\frac{\partial {Y}_{2}}{\partial \xi } = Pe{Y}_{2}$$

### Time transformation to simplify the PDE

A log-time reparameterization was subsequently performed to further simplify the PDE as follows:

21 $$\theta =-ln\left(1+\alpha \tau \right)$$

Substituting $\theta$ in Eq. (19) yields (Appendix B):

22 $$\frac{\partial {Y}_{i}}{\partial \theta } = -\frac{G}{ \alpha }\left[\frac{{\partial }^{2}{Y}_{i}}{\partial {\xi }^{2}}+\left(\frac{2}{\xi }-Pe\xi \right)\frac{\partial {Y}_{i}}{\partial \xi }\right]$$

This remapping is significant because it absorbs the algebraically time-dependent term $G/(1+ \alpha \tau )$ in Eq. (19) into a constant coefficient $-G/\alpha$, yielding a more uniformly scaled PDE over time. The time dependence does not generally disappear; it is simply absorbed by this mapping.

### Logarithmic transformation of the solution variable for the stiff PDE

A significant challenge in solving the transformed drying PDE is the structure of the solute mass fraction field, Y(ξ,θ). As shown in Fig. 1a and expected from the drying physics, Y spans a wide range from a small initial value to much larger near-surface values at late drying times. At the same time, the solution develops steep gradients near the droplet surface as the locking point is approached. These features are difficult for PINNs to represent accurately, because the learned solution may become overly smooth in thin boundary-layer regions, leading to reduced near-surface accuracy and larger discrepancies in stiff regimes^13,34^. To mitigate these difficulties, we introduced the logarithmic transformation

23 $$q\left(\xi ,\theta \right)=\mathrm{ln}\left(Y\left(\xi ,\theta \right)\right)$$

This transformation is useful in the present problem (Fig. 1) because it first compresses the dynamic range of the solution. For example, a *Y* range of [0.001, 0.6] is transformed into a more manageable *q* range of [-6.9, -0.51]. Second, and more importantly, it flattens the steep exponential-like gradients at the boundaries. Exponential growth in the* Y*-space becomes a more linear and smoother function in the* q*-space, which is expected to be easier for the neural network to approximate.

To implement this formulation, the governing equation and BCs were rewritten in terms of the transformed variable *q*. Using the inverse relation $Y = {e}^{q}$, the required derivatives are obtained using the chain rule:

24 $$\frac{\partial Y}{\partial \theta }=\frac{\partial \left({e}^{q}\right)}{\partial \theta } = {e}^{q} \frac{\partial q}{\partial \theta } = Y\frac{\partial q}{\partial \theta }$$

25 $$\frac{\partial Y}{\partial \xi }=\frac{\partial \left({e}^{q}\right)}{\partial \xi }=\left({e}^{q}\right)\frac{\partial q}{\partial \xi }=Y\frac{\partial q}{\partial \xi }$$

26 $$\frac{{\partial }^{2}Y}{\partial {\xi }^{2}}=\frac{\partial }{\partial \xi }\left(Y\frac{\partial q}{\partial \xi }\right)=\left(\frac{\partial Y}{\partial \xi }\right)\left(\frac{\partial q}{\partial \xi }\right)+Y\frac{{\partial }^{2}q}{\partial {\xi }^{2}}=(Y\frac{\partial q}{\partial \xi })(\frac{\partial q}{\partial \xi }) +Y\frac{{\partial }^{2}q}{\partial {\xi }^{2}} = Y\left[{\left(\frac{\partial q}{\partial \xi }\right)}^{2}+\frac{{\partial }^{2}q}{\partial {\xi }^{2}}\right]$$

Substituting these values into Eq. (22) yields:

27 $$Y\frac{\partial q}{\partial \theta }=-\frac{G}{\alpha }\left[Y\left({\left(\frac{\partial q}{\partial \xi }\right)}^{2}+\frac{{\partial }^{2}q}{\partial {\xi }^{2}}\right)+\left(\frac{2}{\xi }-Pe\xi \right)\left(Y\frac{\partial q}{\partial \xi }\right)\right]$$

Factoring out $Y$ results in:

28 $$\frac{\partial q}{\partial \theta }=-\frac{G}{\alpha }\left[\frac{{\partial }^{2}q}{\partial {\xi }^{2}}+\left(\frac{2}{\xi }-Pe\xi \right)\frac{\partial q}{\partial \xi }+{\left(\frac{\partial q}{\partial \xi }\right)}^{2}\right]$$

The IC and BCs were similarly transformed.

29 $$q\left(\xi ,0\right)=ln\left({Y}_{0}\right)={q}_{0}$$

center BC:

30 $$\left( {Y\frac{\partial q}{{\partial \xi }}} \right)\left. \right|_{\xi = 0} = 0 \Rightarrow \frac{\partial q}{{\partial \xi }}\left. \right|_{\xi = 0} = 0$$

surface BC:

31 $$\left( {Y\frac{\partial q}{{\partial \xi }}} \right)\left. \right|_{\xi = 1} - PeY\left. \right|_{\xi = 1} = 0 \Rightarrow Y\left( {\frac{\partial q}{{\partial \xi }} - Pe} \right)\left. \right|_{\xi = 1} = 0$$

This transformation converts Robin BC into a Neumann BC for q, as follows:

32 $$\frac{\partial q}{{\partial \xi }}\left. \right|_{\xi = 1} - Pe = 0\quad \to \quad \frac{\partial q}{{\partial \xi }}\left. \right|_{\xi = 1} = Pe$$

In the transformed surface BC, *Pe* appears as the coefficient controlling the prescribed gradient in *q*-space. The logarithmic transformation changes the original Robin-type condition, which couples *Y* and *∂Y/∂ξ*, into a simpler Neumann condition for *q*. For a fixed operating condition, this boundary target is constant in space and time, which simplifies the surface residual evaluated during PINN training.

Thus, the logarithmic transformation is used here as a physics-guided reformulation rather than an arbitrary preprocessing step. Unlike simple linear normalization or output scaling, it both compresses multiplicative variations in *Y*-space and simplifies the surface BC in the transformed formulation.

Because *q* = *lnY* is an auxiliary transformed variable, all final predictions are mapped back to the physical mass fraction using *Y* = *exp(q)*. Therefore, the logarithmic transformation does not change the physical interpretation of the solution; it only changes the variable learned during training. In addition, small errors in q correspond approximately to relative errors in Y, which is advantageous when the solution spans a wide dynamic range.

## Reference Crank–Nicolson (CN) solution

The superheated-steam drying model discussed in the previous sections was also solved using an implicit CN method. The CN discretization was constructed for the same *Y*-space governing equation used by the baseline PINN, with conservative spatial discretization and second-order boundary elimination at both the center and surface boundaries. The reference computations used 1601 grid points in the spatial domain and 6400 uniform steps in the transformed time variable.

Because the CN solution is a numerical approximation rather than an exact analytical solution, its reliability as a benchmark was assessed separately through a dedicated CN verification study. This verification included (i) a semi-discrete spectral analysis together with an assessment of the CN amplification factors, (ii) a manufactured-solution local truncation-residual study as consistency evidence, (iii) a coupled space–time refinement study based on the method of manufactured solutions (MMS) for convergence assessment, and (iv) an actual-problem grid-refinement study. These checks follow standard numerical verification practices, including matrix method stability analysis, manufactured solution truncation error monitoring, systematic MMS order-of-accuracy verification, and grid convergence analysis ^35–38^.

The verification results showed bounded CN amplification for the examined dissipative modes, decreasing truncation residuals under refinement, MMS convergence consistent with the expected second-order behavior, and reduced grid sensitivity for the actual problem, with the highest superheating case remaining the most demanding (Appendix C). Accordingly, the agreement between the PINN and CN should be interpreted as an agreement with a numerically verified reference benchmark, and not with an exact analytical solution. This numerical benchmarking was intended to assess the formulation accuracy under controlled conditions and should not be interpreted as a substitute for the experimental validation of the underlying drying process.

The material selected here was an SiO₂-water nanosuspension, as in Bück^2^; Table 1 presents the material properties.

**Table 1:** Material parameters of the liquid and dispersed solids. The liquid data correspond to the boiling point.

Parameter/Unit	Value
Mass density (liquid water) /${kg/m}^{3}$	958.3
Mass density $\left({SiO}_{2}\right)/{kg/m}^{3}$	2210.0
Specific evaporation enthalpy/$J/kg$	2,250,000
Dynamic viscosity (liquid water) /$Pa s$	$0.2822\times {10}^{-3}$
Initial droplet radius / m	$0.5\times {10}^{-3}$
Initial solid concentration /${m}^{3}/{m}^{3}$	0.001
Nanoparticle radius / m	$20\times {10}^{-9}$
Boiling temperature (water) / K	372.15
Nusselt number / -	2
Superheating temperature / K	10–100
Boltzmann constant (${{\boldsymbol{k}}}_{{\boldsymbol{B}}}$) /$J/K$	$1.3806\times {10}^{-23}$

## Implementation of PINNs

Physics-informed neural networks were used to solve the superheated steam drying problem and determine the solute concentration profiles within droplets. Physics-informed neural networks use fully connected feedforward neural networks to solve equations and consist of multiple hidden layers. In this study, the PINN takes $\theta$ and ${\xi }^{2}$ as inputs and provides $Y$ (or *q* in the case of logarithmically transformed models) as the output. The use of the squared coordinate, ${\upxi }^{2}$, enforces physical symmetry, guaranteeing that the spatial derivative vanishes at the center by construction. This architectural choice effectively removes the need for an explicit center BC in the loss function and simplifies the training process, as established in our previous study^12^. The weights and biases were updated during training until the optimal parameters were obtained^4^.

To demonstrate the severity of the gradient stiffness in superheated steam drying, we established a baseline model using a standard PINN backbone. This model approximates the mass fraction $Y\left(\upxi ,\uptheta \right)$ directly, without logarithmic transformation.

### Loss functions and BC residual scaling

In the standard baseline PINN, the loss terms are minimized during training to penalize deviations in the dynamics of the PDE and surface BC, respectively:

33 $${Loss}_{PDE}= \frac{1}{{N}_{PDE}}\sum_{j=1}^{{N}_{PDE}}{\left(\frac{\partial {Y}_{2}}{\partial \theta }+\frac{G}{ \alpha }\left[\frac{{\partial }^{2}{Y}_{2}}{\partial {\xi }^{2}}+\left(\frac{2}{\xi }-Pe\xi \right)\frac{\partial {Y}_{2}}{\partial \xi }\right]\right)}^{2}$$

34 $${Loss}_{surface}=\frac{1}{{N}_{surface}}\sum_{k=1}^{{N}_{surface}}{\left({\left.\left(\frac{\partial {Y}_{2}}{\partial \xi }-Pe{Y}_{2}\right)\right|}_{\xi =1}\right)}^{2}$$

where ${N}_{PDE}$ and ${N}_{surface}$, denote the number of collocation points used to enforce the PDE residual and the surface BC, respectively. The indices *j* and *k* enumerate these collocation points in their corresponding sets. The IC is hard-encoded as follows:

35 $$Y\left(\xi ,\theta \right)={Y}_{0}+\left(1-{e}^{-\theta }\right)\cdot \mathcal{N}\left(\xi ,\theta \right)$$

where ${Y}_{0}$ is the known IC value. The term $\mathcal{N}\left(\xi ,\theta \right)$ represents the output of the neural network. The factor $\left(1-{e}^{-\theta }\right)$ serves as a temporal distance function that vanishes at $\theta$ = 0. Therefore, at the initial time, the network contribution is switched off, and the solution is forced to equal the prescribed initial value everywhere in the domain. As time advances ($\theta$ > 0), this factor increases from 0 to 1, gradually activating the network output so that the model learns the departure from the initial state and the subsequent temporal evolution^39^. Using this construction, the network learns the temporal evolution as a correction to the initial state, eliminating the need for an explicit IC loss term.

Combining these loss terms (Eqs. (33) and (34)), the total loss function is defined as follows:

36 $${Loss}_{total} = {{\omega}_{PDE}Loss}_{PDE}+ {\omega}_{BC, surface}{Loss}_{surface}$$

where $\omega$ denotes the weights of the respective loss components.

For the scaled LT-PINN, the surface BC residual was scaled based on the *Pe*, as follows:

37 $${Loss}_{PDE}= \frac{1}{{N}_{PDE}}\sum_{j=1}^{{N}_{PDE}}{\left(\frac{\partial {q}_{2}}{\partial \theta }+\frac{G}{ \alpha }\left[\frac{{\partial }^{2}{q}_{2}}{\partial {\xi }^{2}}+\left(\frac{2}{\xi }-Pe\xi \right)\frac{\partial {q}_{2}}{\partial \xi }+{\left(\frac{\partial {q}_{2}}{\partial \xi }\right)}^{2}\right]\right)}^{2}$$

38 $${Loss}_{surface}=\frac{1}{{N}_{surface}}\sum_{k=1}^{{N}_{surface}}{\left(\left(\frac{1}{Pe+\varepsilon }\right)\left(\frac{\partial {q}_{2}}{\partial \xi }-Pe\right){\left.\right|}_{\xi =1}\right)}^{2}$$

39 $$q\left(\xi ,\theta \right)={ln(Y}_{0})+\left(1-{e}^{-\theta }\right)\cdot \mathcal{N}\left(\xi ,\theta \right)$$

The BC residual scaling in Eq. (38) is introduced because the transformed BC contains an explicit *Pe*-dependent residual. Without scaling, the magnitude of this residual can increase approximately with *Pe*, and its squared contribution to the loss can therefore grow approximately as *Pe*^*2*^, particularly before the BC is well satisfied. This can cause the surface term to dominate the optimization and reduce the relative influence of the interior PDE residual.

To reduce this Pe-driven imbalance, the surface residual is multiplied by *1/(Pe* + *ε)*, with *ε* = *10*^*−6*^. This keeps the boundary-loss contribution more comparable across operating conditions while preserving the physical form of the transformed BC. Thus, the proposed scaling is not an arbitrary weighting choice, but a physics-informed normalization of the *Pe*-dependent surface residual. The LT-PINN uses the same transformed PDE and IC construction, but without the *1/(Pe* + *ε)* scaling in the surface loss.

**Fig. 2:**
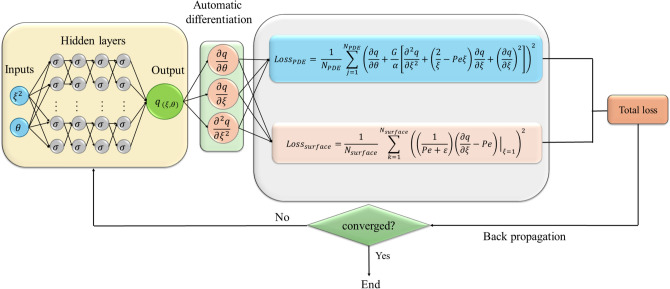
Schematic of the training pipeline of the proposed scaled LT-PINN formulation. The inputs *θ* and *ξ*^*2*^ are passed through a neural network to predict the transformed variable *q(ξ,θ)*. Subsequently, automatic differentiation was used to construct the transformed PDE and surface boundary losses, and the surface residual was scaled by *1/(Pe* + *ε)* before forming the total loss for backpropagation.

An overview of the scaled LT-PINN formulation is shown in Fig. 2, which illustrates the automatic differentiation operators and surface loss derived from the *Pe*-scaled BC residual used to construct the total loss function.

### PINN configuration and training settings

The details of the baseline PINN configuration and training settings are summarized in Table 2. The network depth and width were selected using a systematic grid search method. We defined a discrete search space spanning network depths of $L\in 2...5$ hidden layers and widths of $N\in {2}^{\mathrm{k}}, k=5, 6, 7, 8$ neurons per layer. For each configuration pair $\left(L,N\right)$, the model was trained and evaluated using a validation metric computed relative to the CN numerical reference solution. The selected network comprised two hidden layers with 128 neurons per hidden layer, followed by a linear output layer. This architecture was fixed for all the compared formulations. The PINN predictions were evaluated against the CN numerical reference solution over superheating temperatures ranging from 10 to 100 K, covering cases from relatively uniform to strongly steepened concentration profiles. At each evaluation ${\theta}_{k}$​, the absolute relative difference (ARE) between the PINN prediction and the CN reference solution was computed over the spatial grid as

40 $$ARE\left({\theta}_{k}\right)=\frac{1}{{N}_{x}}\sum_{j = 1}^{{N}_{x}}\frac{\left|{Y}_{PINN}\left({\xi}_{j},{\theta}_{k}\right)-{Y}_{CN}\left({\xi}_{j},{\theta}_{k}\right)\right|}{{Y}_{CN}\left({\xi}_{j},{\theta}_{k}\right) + \varepsilon }$$

where ${N}_{x}$​ is the number of spatial evaluation points, and ε is a small constant to prevent division by zero. The reported field metric was defined as the mean absolute relative error (field MARE), which was obtained by averaging this quantity over all *K* evaluation times used in the CN–PINN comparison, as follows:

**Table 2:** PINN configuration and training settings.

Item	Setting
Network	MLP with 2 hidden layers, 128 neurons per hidden layer, and 1 linear output layer
Activation function	Tanh (all hidden), last layer linear
Random seeds (reproducibility)	42, 101, 1234, 2024, 5678
Initialization	Xavier/Glorot normal for weights, zeros for biases
Collocation sampling	${N}_{PDE}=\mathrm{2,000}, {N}_{surface}=\mathrm{1,000}$
Optimizer	Hybrid strategy: Adam at an initial learning rate of 0.001 with a learning rate scheduler
Epochs / resampling	Adam (Max 10,000) + L-BFGS (max 500 iterations); resample every 500 epochs
Early stopping	Patience = 500 epochs, threshold $\updelta ={10}^{-5}$ (Adam phase only)
Loss metrics	Mean squared error (MSE) of the residuals
Software & libraries	PyTorch 2.1.0 (Python 3.9.18) on CUDA 11.8 / cuDNN 8.7
Device	GPU: NVIDIA RTX A4500 (20 GB)

41 $$Field MARE= \frac{100}{K}\sum_{k = 1}^{K}ARE\left({\theta}_{k}\right)$$

The training process employed a hybrid optimization strategy that combined the robustness of Adam with the high-precision convergence of L-BFGS. To optimize the computational resources, we implemented an adaptive early stopping mechanism during the Adam phase. The training loop monitored the total loss and terminated the Adam optimizer if the loss improvement failed to exceed a threshold of $\delta ={10}^{-5}$ for 500 consecutive epochs (patience).

This criterion serves as a plateau detection mechanism, indicating that the first-order optimizer has reached the limit of its effective convergence rate. Upon satisfying this condition (or reaching the maximum budget of 10,000 epochs), the training immediately transitioned to the L-BFGS optimizer (second-order quasi-Newton method) to fine-tune the solution weights to machine precision. This dual-phase approach ensures that the model does not stagnate in shallow local minima while avoiding excessive computational overhead with diminishing returns.

To systematically isolate the contributions of our proposed improvements (log transformation and BC residual scaling), we conducted a rigorous three-step ablation study. We defined three incremental formulations, all using the same PINN backbone, to test the hypothesis that standard PINN formulations fail because of the specific combination of exponential growth and stiff BCs:

1. Baseline (Step 1): A standard PINN formulation that operates directly in the physical domain (*Y*-space) and serves as the control formulation.
2. LT-PINN (Step 2): The physics-guided use of the logarithmic transformation $q=\mathrm{ln}\left(Y\right)$, motivated by the known wide dynamic range and steep near-surface growth of the solution, while retaining the standard loss weighting.
3. Scaled LT-PINN (Step 3): The complete method integrates both the logarithmic transformation and BC residual scaling.

To ensure statistical robustness and rule out the influence of favorable weight initializations, each configuration was trained independently across five distinct random seeds (S ∈ {42, 101, 1234, 2024, 5678}). All performance metrics reported in this study are presented as mean ± standard deviation over these independent trials. This protocol confirms that the reported stability improvements result from the proposed formulation, rather than stochastic variance.

To examine the training imbalance following Wang et al.^20^, we also monitored the gradient norm associated with each loss component during optimization. For each loss component Loss_i_​, we report the Euclidean norm of its parameter gradient,

42 $${\Vert {\nabla}_{w}{Loss}_{i}\Vert }_{2} = \sqrt{\sum_{p}({\frac{\partial {Loss}_{i}}{\partial {w}_{p}})}^{2}}$$

evaluated using the same diagnostic batch used for the corresponding loss values. Large disparities in gradient norms across loss terms indicate that one component exerts a disproportionate influence on parameter updates and, therefore, dominates the training.

To assess the computational cost, we recorded the computational times of the best-performing PINN model and CN reference solver on the same hardware. For the PINN, we report both the total training time (Adam + L-BFGS) and the post-training prediction time, whereas for the CN, we report the time required for one numerical solution.

To verify that the poor performance of the baseline PINN was not due to insufficient tuning, we conducted an additional baseline robustness study. This study systematically explored the baseline architecture, optimizer settings, early stopping strategy, and collocation densities in staged sweeps, starting from the hardest case and then extending the strongest candidates to broader multi-seed evaluations. Unless otherwise stated, the robustness runs used the same Adam budget as the main study (up to 10,000 epochs). Across these tests, the baseline PINN remained unable to reliably recover the locking event, indicating that its poor performance was primarily formulation-related rather than due to weak hyperparameter tuning. A compact summary of this study is provided in Appendix D (Table 4).

### Alternative PINN improvement strategies

In addition to the three main formulations described above, we considered several representative directions for improving PINNs for comparative analysis. These additional tests were not intended to be exact reproductions of the original literature methods but rather controlled comparator variants designed to assess whether generic PINN refinements could either rescue the direct *Y*-space baseline or replace the proposed BC residual scaling once the logarithmic state transformation was already applied.

Four comparison directions were examined. First, we considered a linearly scaled *Y*-space PINN, which used the same (ξ^2^, θ) input representation as the other PINNs but linearly rescaled the network output before mapping it back to *Y*. This was performed to test whether simple output-range normalization alone can rescue the direct *Y*-space formulation. Second, we considered a gradient-based adaptive weighting variant motivated by prior work on balancing PINN loss components using gradient information^20^. In this variant, the relative influence of the PDE and BC loss terms was adjusted during training based on the magnitudes of their gradients. Third, we considered a surface-biased collocation variant motivated by sampling-based PINN strategies that place greater emphasis on training in difficult regions of the domain^9,15^. In the present benchmark, this bias was implemented by redistributing a prescribed fraction of PDE collocation points toward the near-surface region, where the boundary layer is steepest. Finally, we adopted a stronger architectural baseline by using a deeper and wider multilayer perceptron instead of a shared reference network.

These additional strategies were applied in two complementary settings. First, the selected refinements were tested as possible rescue mechanisms for a direct *Y*-space baseline formulation. Second, they were tested in combination with the logarithmic state transformation but without the proposed Pe-based BC residual scaling to examine whether these methods could replace the proposed scaling. Accordingly, the purpose of these comparisons was not to claim superiority over all published PINN improvements but to assess whether representative refinements can match the benefits of the proposed physics-guided formulation in the present benchmark.

## Results and discussion

### Validation of PINN formulations against the numerically verified CN reference

Table 3 presents the field MARE, surface discrepancy at ξ = 1, and locking-time relative difference for the models (baseline PINN, LT-PINN, and scaled LT-PINN) for *ΔT* = 10–100 K. All discrepancies reported in this section were measured with respect to the numerically verified CN reference solution.

**Table 3:** Accuracy and locking-time differences relative to the CN reference solution for the ablation study across superheating levels.

Superheating temperature (K)	Model	Field MARE (%)	Surface discrepancy relative to CN (%)	Locking-time relative difference from CN (%)
10	Baseline	96.96 ± 0.90	5.24 ± 0.01	Failed
	LT-PINN	1.01 ± 0.95	0.09 ± 0.07	0.04 ± 0.04
	Scaled LT-PINN	2.16 ± 1.43	0.20 ± 0.13	0.07 ± 0.08
30	Baseline	97.34 ± 0.54	7.70 ± 0.00	Failed
	LT-PINN	1.18 ± 0.45	0.23 ± 0.08	0.06 ± 0.15
	Scaled LT-PINN	1.20 ± 0.70	0.24 ± 0.15	0.11 ± 0.08
50	Baseline	97.13 ± 0.79	9.49 ± 0.00	Failed
	LT-PINN	3.14 ± 1.18	1.43 ± 0.85	0.68 ± 0.39
	Scaled LT-PINN	1.58 ± 0.59	0.86 ± 0.36	0.26 ± 0.13
70	Baseline	97.09 ± 0.74	10.93 ± 0.00	Failed
	LT-PINN	3.56 ± 1.12	2.37 ± 1.15	3.65 ± 1.98
	Scaled LT-PINN	1.32 ± 0.48	0.80 ± 0.35	0.04 ± 0.02
100	Baseline	97.20 ± 1.09	12.67 ± 0.00	Failed
	LT-PINN	4.53 ± 1.22	4.64 ± 1.95	4.25 ± 0.29
	Scaled LT-PINN	2.19 ± 1.15	1.16 ± 1.39	1.61 ± 0.73

Despite apparently moderate surface discrepancies at low superheating, the baseline PINN exhibited an approximately constant ∼97% field MARE across all superheating temperatures and failed to predict the locking time. This is best interpreted as an optimization failure rather than a lack of training time. The model collapses to an overly smooth, near-trivial function that does not capture the sharp near-surface boundary layer growth and, therefore, cannot reproduce the threshold-based locking event.

**Fig. 3:**
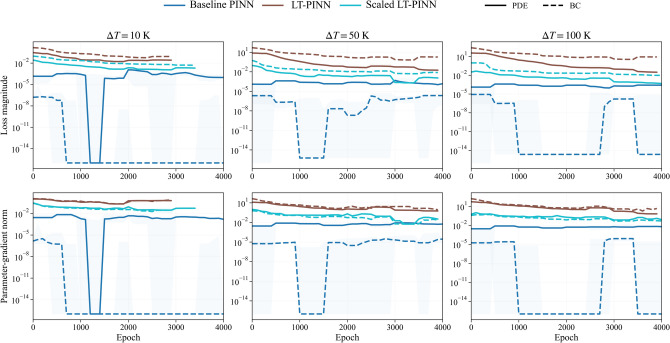
Training diagnostics for the baseline PINN, LT-PINN, and scaled LT-PINN at representative superheating levels (ΔT = 10, 50, and 100 K). Top row: PDE and BC loss. Bottom row: Norms of the corresponding parameter gradients. Solid and dashed lines denote the PDE and BC terms, respectively. The curves show the median over five random seeds, and the shaded bands indicate the interquartile range.

This interpretation is supported by the training diagnostics in Fig. 3, which report the PDE and BC losses, together with the corresponding parameter–gradient norms. Rather than achieving a stable joint minimization of the PDE and BC objectives, the baseline PINN enters a pathological training regime in which the BC-related signal becomes extremely weak, whereas the predicted solution remains inaccurate. Thus, a low training loss should not be interpreted as successful convergence. This interpretation is consistent with the oversmoothed spatial profiles and the persistent failure to reach locking, as shown in Fig. 5a. For completeness, the total loss convergence histories are provided in the Supplementary Material (Fig. S1).

The introduction of *q* = *ln Y* (LT-PINN) yields a substantial improvement. The field MARE decreases from ∼97% to 1.01–4.53%, and the locking time becomes predictable with a very small relative difference with respect to the CN reference at mild stiffness (0.04 ± 0.04% at *ΔT* = 10 K and 0.06 ± 0.15% at *ΔT* = 30 K). These gains reflect two effects: (i) dynamic-range compression reduces saturation and makes gradients learnable, and (ii) the Robin BC becomes a constant Neumann condition, which removes the direct value–gradient coupling at the boundary.

However, the LT-PINN performance degrades systematically at higher *ΔT*, which corresponds to a higher *Pe* in the present benchmark. This deterioration is linked to the increasing dominance of convection over diffusion at higher *Pe*. This behavior is also reflected in the diagnostics shown in Fig. 3. As *Pe* increases, the near-surface layer becomes thinner and steeper, increasing the sensitivity of the LT-PINN to boundary-loss imbalance. The surface discrepancy increased from 0.09 ± 0.07% at *ΔT* = 10 K to 4.64 ± 1.95% at *ΔT* = 100 K, and the locking-time difference relative to the CN reference increased to 4.25 ± 0.29% at *ΔT* = 100 K. Even with the simplified Neumann BC, the magnitude of the boundary residual scales with *Pe* (Eq. (32)). Therefore, the squared residual contribution (loss) scales approximately as *Pe*^*2*^. Consequently, increasing *Pe* shifts the optimization toward stronger BC dominance in the unscaled LT-PINN, reducing the network’s ability to jointly minimize the interior PDE residual and preserve accurate near-surface gradients at late times.

The proposed scaled LT-PINN introduces a *Pe*-aware scaling (division by (*Pe* + *ε*) in the surface residual). As shown in Fig. 3, this scaling produced more stable loss and gradient behavior across the full *ΔT* range by reducing the temperature dependence of the boundary-loss imbalance. Quantitatively, the field MARE remained below approximately 2.2% at all superheating temperatures. The improvement was most significant in stiffer regimes. At *ΔT* = 50 K, the surface discrepancy decreased from 1.43 ± 0.85% to 0.86 ± 0.36%; at *ΔT* = 70 K, from 2.37 ± 1.15% to 0.80 ± 0.35%; and at *ΔT* = 100 K, from 4.64 ± 1.95% to 1.16 ± 1.39%. Likewise, the relative difference in the locking time was reduced from 3.65 ± 1.98% to 0.04 ± 0.02% at *ΔT* = 70 K and from 4.25 ± 0.29% to 1.61 ± 0.73% at *ΔT* = 100 K.

Overall, Table 3 indicates that the logarithmic transformation is the primary step that makes the stiff solution representable and trainable, whereas *Pe*-based boundary scaling prevents stiffness-induced loss imbalances from degrading the surface accuracy and locking-point prediction in convection-dominated regimes.

Physically, the advantage of the scaled LT-PINN becomes most apparent when drying is governed by a thin near-surface boundary layer. This suggests that a similar physics-guided scaling may be useful for other moving-boundary PDEs, in which a dominant interface constraint and a clear stiffness indicator govern the solution quality.

A timing comparison was also performed between the best-performing PINN variant and the CN reference solver. Averaged across all tested temperatures, the training time of the proposed PINN was 45.33 s, whereas a single CN solution required 10.17 s. Thus, the proposed PINN is more expensive than the CN in terms of one-time offline computation. However, once trained, the model provides millisecond-level predictions and can be interpreted as an offline-trained surrogate model for repeated evaluations. Accordingly, the main computational value of the proposed approach lies in fast post-training prediction rather than reducing the cost of a single direct numerical solution.

### Comparison with alternative PINN improvement strategies

**Fig. 4:**
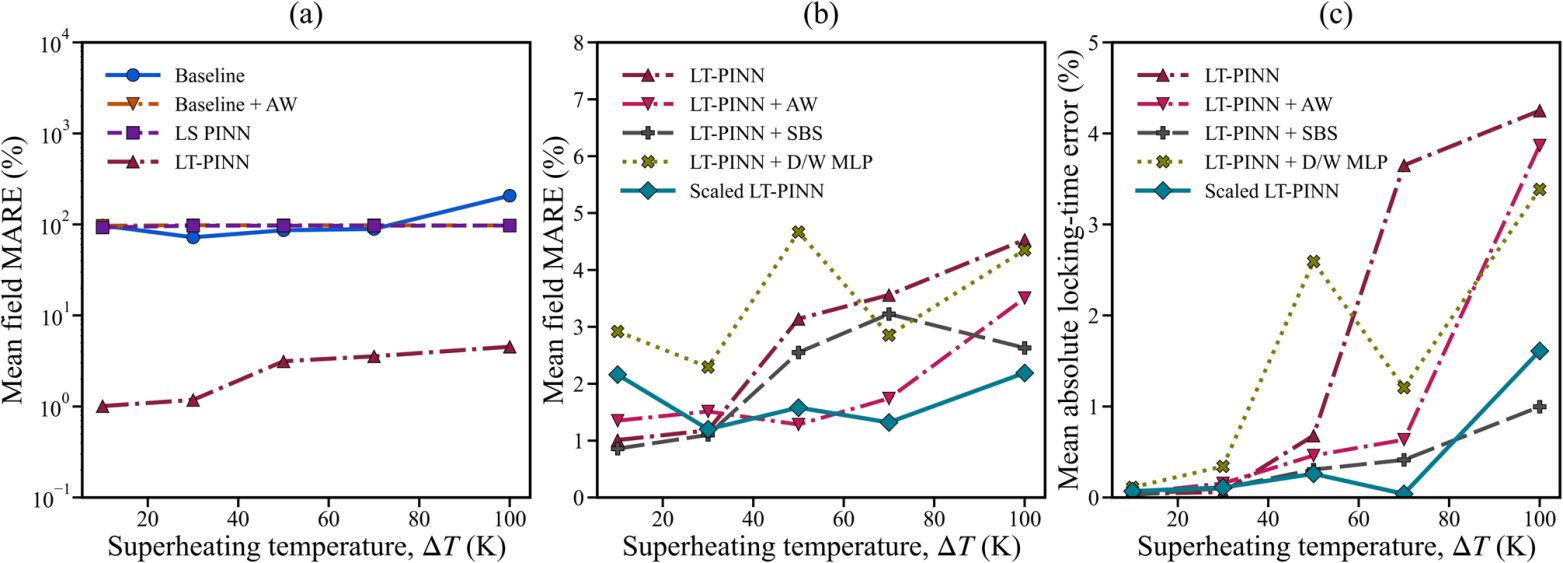
Comparison with representative PINN refinements. (**a**) Field MARE for selected *Y*-space rescue variants with LT-PINN included as a reference. (**b**) Field MARE for LT-PINN formulations. (**c**) Mean absolute locking-time error for LT-PINN formulations. Abbreviations: AW, adaptive weighting; SBS, surface-biased sampling; D/W MLP, deeper/wider multilayer perceptron; LS PINN, linearly scaled *Y*-space PINN.

Figure 4 compares the proposed formulation with several representative general PINN refinements. The purpose of this comparison is not to claim superiority over all published implementations but to assess whether common improvement directions can either rescue the original *Y*-space formulation or replace the proposed BC residual scaling once the logarithmic transformation has been applied.

Panel (a) shows that the representative refinements applied directly in the *Y*-space do not resolve the dominant failure mode of the present benchmark. The baseline PINN, adaptive weighting *Y*-space variant, and linearly scaled *Y*-space variant all remained at very large field MARE levels across the investigated superheating range. In contrast, the logarithmically transformed formulation (LT-PINN) reduced the field error by more than an order of magnitude and yielded physically meaningful predictions throughout the tested range. This comparison indicates that the main difficulty is not removed by generic training refinements or simple output scaling alone but is strongly tied to the original choice of dependent-variable representation.

Panels (b) and (c) examine whether generic refinements can replace the proposed *Pe*-based BC residual scaling after logarithmic transformation. In terms of field accuracy, all LT-PINN-based variants performed substantially better than the non-transformed models, confirming that the logarithmic reformulation is the main step that makes the problem learnable. However, the unscaled LT-PINN and its generic variants generally showed stronger deterioration as the superheating level increased. The scaled LT-PINN was not uniformly the best in every low- or moderate-stiffness case; nevertheless, it provided the most favorable full-field behavior in the high-stiffness regime. In particular, at the highest superheating level, it achieved the lowest field MARE among the tested LT-PINN-based variants.

The locking-time results in panel (c) show a more metric-specific behavior. Because locking is a threshold-based quantity evaluated at the droplet surface, surface-targeted refinements can improve this metric without necessarily improving the full concentration field. At low-to-moderate superheating levels, the differences among the LT-PINN-based models remained relatively small. At higher superheating, the locking-time error of the unscaled LT-PINN and some generic *q*-space refinements increased more strongly. The scaled LT-PINN retained competitive locking-time accuracy and clearly improved over the unscaled LT-PINN at high superheating. However, the surface-biased sampling variant produced a smaller locking-time error in some cases, which is expected because it explicitly concentrates collocation points near the surface where the locking criterion is evaluated. This improvement was not accompanied by the same level of full-field accuracy, as shown in panel (b).

Overall, Fig. 4 supports two conclusions. First, the logarithmic transformation is an essential step that converts the present drying problem from an effectively unlearnable *Y*-space formulation into a tractable PINN problem. Second, once this transformation is applied, the proposed BC residual scaling provides an additional formulation-level robustness benefit, especially for full-field accuracy in the high-*Pe* regime. Generic refinements such as adaptive weighting, surface-biased sampling, or a deeper/wider MLP can provide partial or metric-specific improvements, but they do not replace the balanced full-field improvement obtained from the physics-guided scaled LT-PINN formulation.

### Concentration profile validation and boundary-layer resolution

**Fig. 5:**
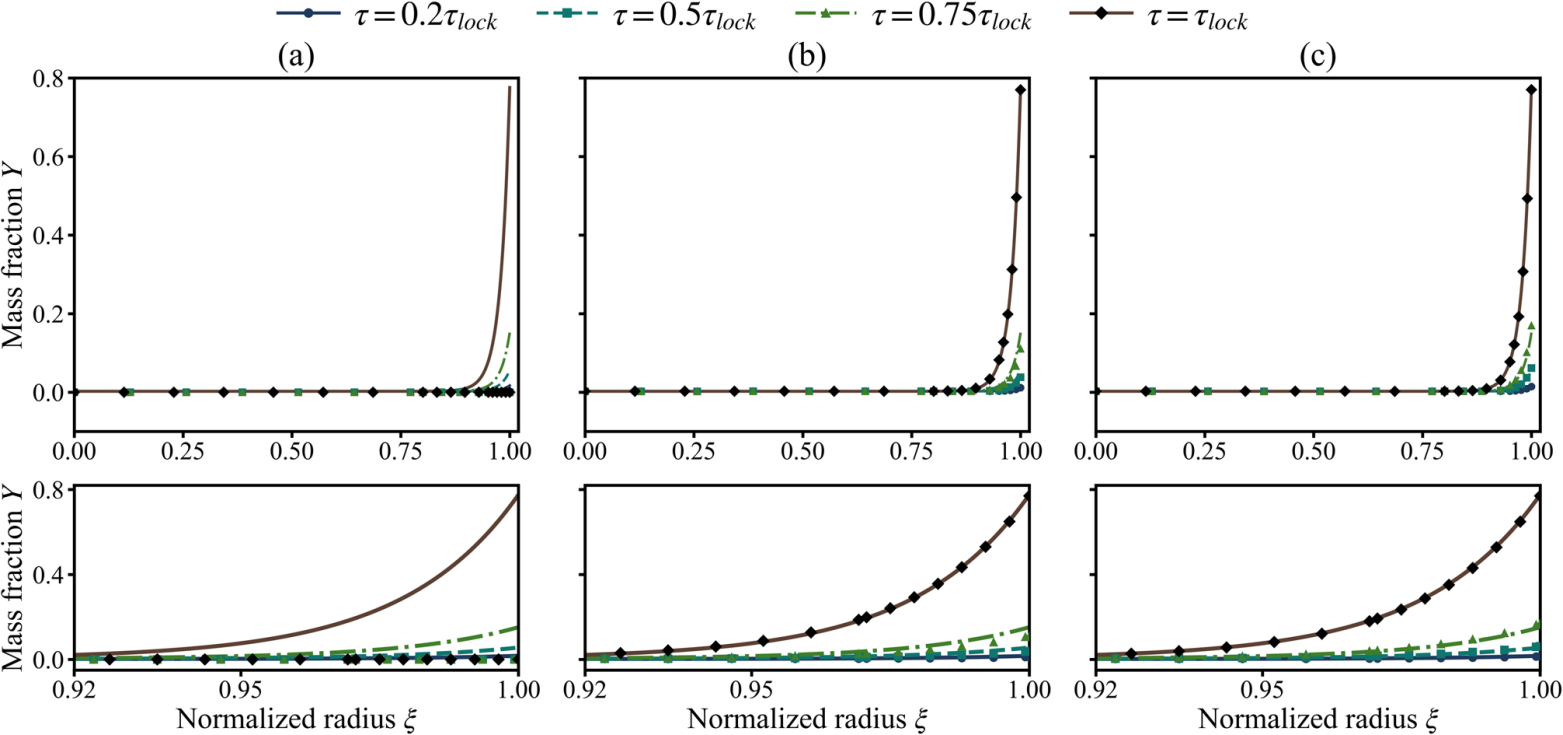
Mass-fraction profiles of (**a**) baseline PINN, (**b**) LT-PINN, and (**c**) scaled LT-PINN versus the CN reference at 100 K for representative times. Top row: Full-domain profiles. Bottom row: Zoomed-in near-surface region.

**Fig. 6:**
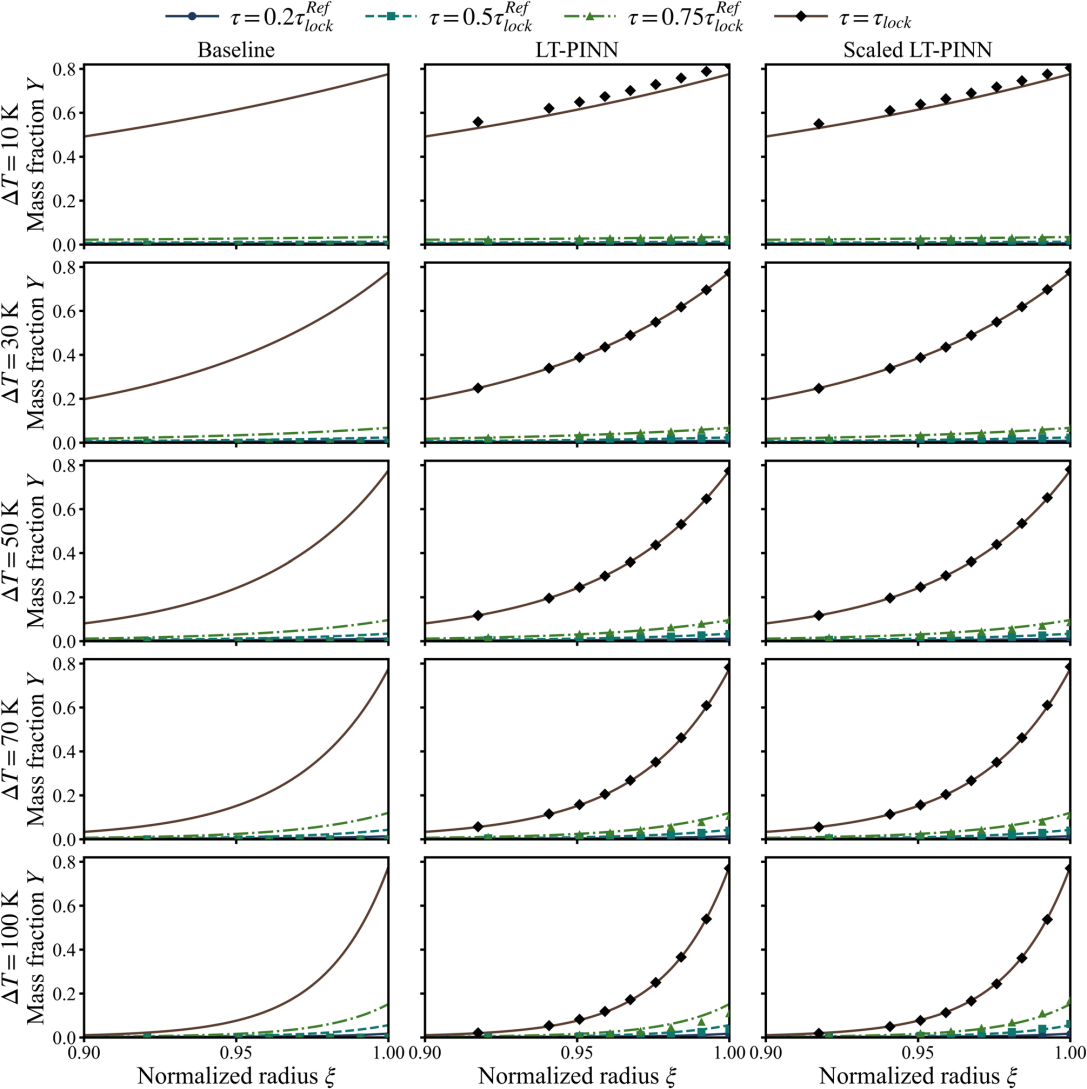
Near-surface mass fraction profiles of baseline PINN, LT-PINN, and scaled LT-PINN vs. CN numerical reference solution at different times for different superheating temperatures.

Figures 5 and 6 compare the predicted mass-fraction profiles with the CN reference at selected spatial and temporal locations. These figures demonstrate that the solution difficulty is non-uniform across the domain. As demonstrated in these figures, the difficulty is concentrated near ξ = 1, particularly at late times. This localization explains why global metrics alone are insufficient and why surface-focused validation is necessary.

In the representative stiff case (100 K, Fig. 5), the baseline PINN collapsed to a near-trivial, overly smooth profile and failed to reproduce the sharp, near-surface gradient. This behavior is consistent with under-resolution of the boundary layer, where a smooth approximation can reduce parts of the training loss without capturing the high-gradient surface structure required by the physics. The LT-PINN recovered the correct qualitative trend (monotonic increase toward the surface) and substantially improved the agreement with the CN reference. This supports the interpretation that the difficulty is strongly linked to the large dynamic range and nonlinear growth of *Y* in the original *Y*-space formulation. The scaled LT-PINN achieved the closest agreement near *ξ → 1*, suggesting that a substantial portion of the remaining LT-PINN error is attributable to boundary-localized optimization imbalance rather than to the representational capacity of the shared PINN backbone alone.

The temperature sweep (Fig. 6) supports this interpretation. As *ΔT* increased, the effective *Pe* also increased in the present drying benchmark; therefore, the problem became more strongly convection-dominated. Consequently, the boundary layer became thinner, and the mass fraction profiles became steeper. The LT-PINN deviations grew predominantly in the near-surface region rather than uniformly across the domain, which is consistent with a regime shift toward boundary-dominated training at high *Pe*. In contrast, the scaled LT-PINN maintained substantially more stable near-surface agreement across the *ΔT* range, supporting the conclusion that BC residual scaling stabilizes learning specifically in the physically dominant surface layer, where locking occurs.

### Surface evolution and locking-time prediction

Locking is a threshold event defined by the surface concentration reaching a critical value; therefore, surface trajectories are decisive in evaluating the usefulness of the model. In stiff cases, the surface concentration increases sharply near the locking, so even a small bias in the predicted surface trajectory can lead to a noticeable shift in the predicted locking time.

**Fig. 7:**
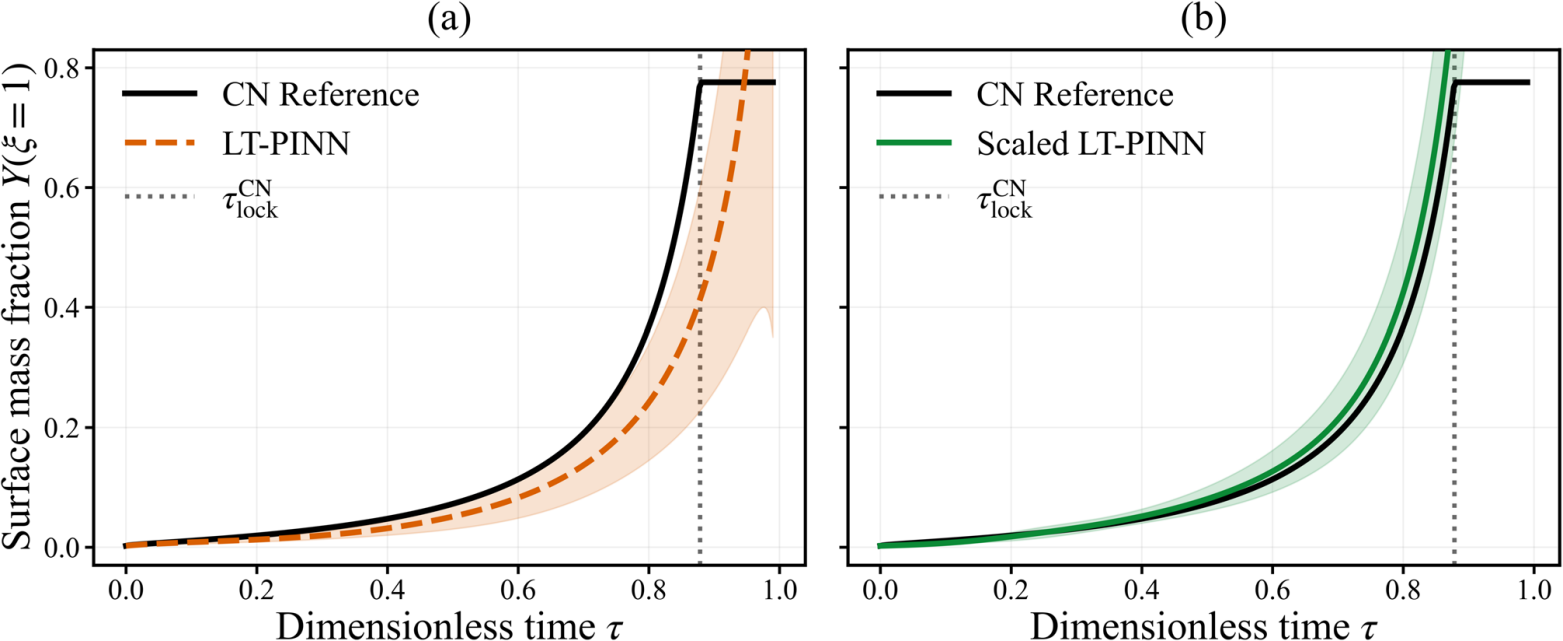
Surface evolution and locking-point prediction of (**a**) LT-PINN and (**b**) scaled LT-PINN at superheating temperature of 100 K. The shaded region denotes the variability across independent training runs (mean ± standard deviation across five runs with different random seeds).

Figure 7 shows that both the LT-PINN and scaled LT-PINN can identify the locking point. However, the LT-PINN exhibited a larger mismatch in the late-time surface rise and the corresponding locking time. The scaled LT-PINN tracked the CN surface evolution more closely, which explains its systematically lower locking-time differences relative to the CN reference under the stiffest conditions (Table 3).

**Fig. 8:**
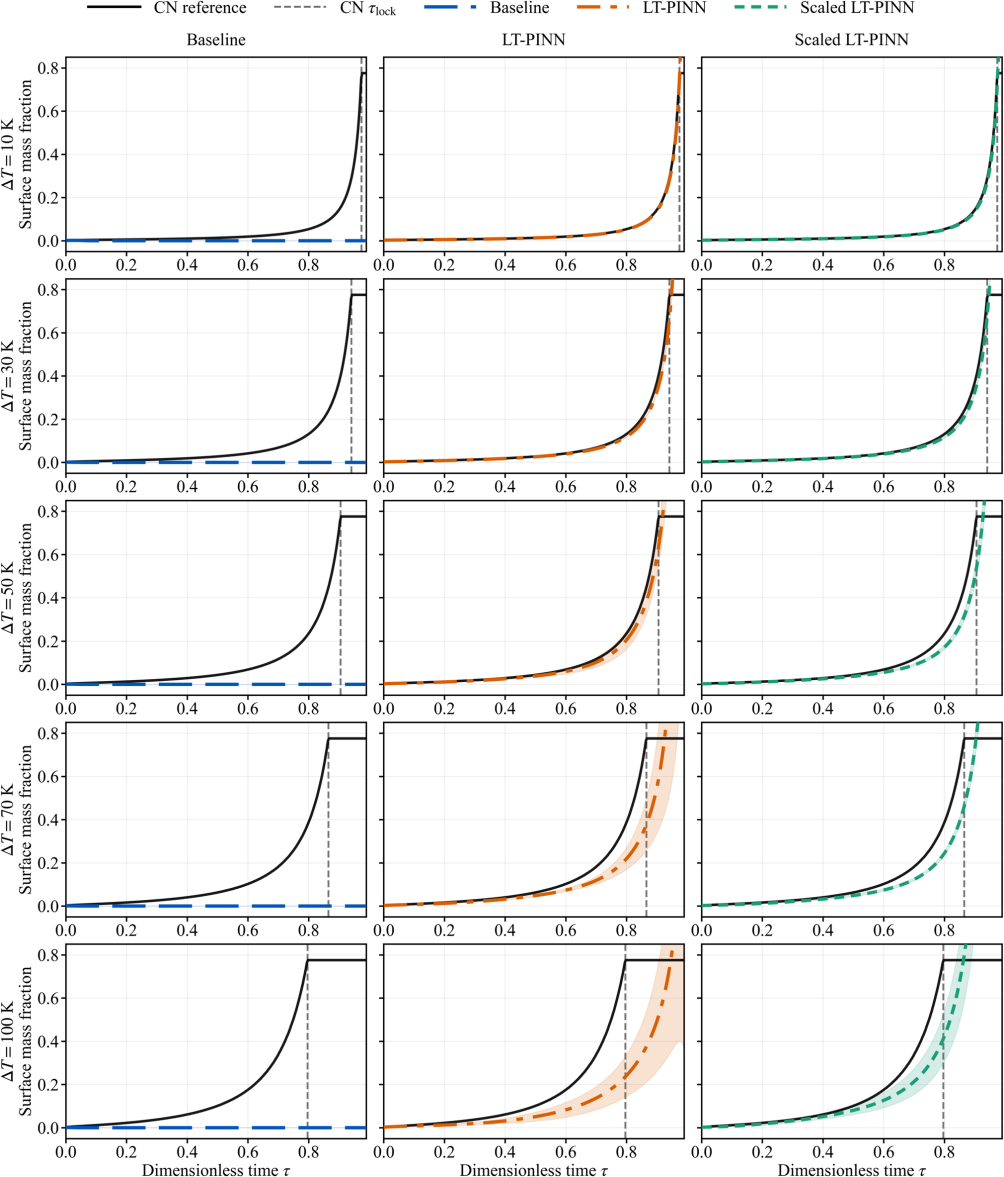
Surface evolution and locking-point prediction of baseline PINN, LT-PINN, and scaled LT-PINN across different superheating temperatures. The shaded region denotes the variability across independent training runs (mean ± standard deviation across five runs with different random seeds).

Figure 8 generalizes this behavior for different *ΔT* values. The baseline PINN remained close to a near-trivial surface trajectory and failed to reach the locking threshold, consistent with its failure to predict locking. As the superheating temperature increased, surface enrichment became faster, and the late-time surface rise became steeper. The LT-PINN captured the overall surface-growth trend but showed increasing deviation from the CN reference at high ΔT. In contrast, the scaled LT-PINN predictions remained close to the CN across the temperature range and showed a clearer advantage near the threshold region at moderate-to-high superheating, where its locking-time differences were lower than those of the unscaled LT-PINN. These results support the engineering implication that, beyond reducing average field discrepancy, *Pe*-based BC residual scaling improves the reliability of threshold-event prediction, which is directly relevant to locking, morphology development, and process prediction in particle engineering.

### CN-referenced discrepancy trends versus superheating and robustness assessment

**Fig. 9:**
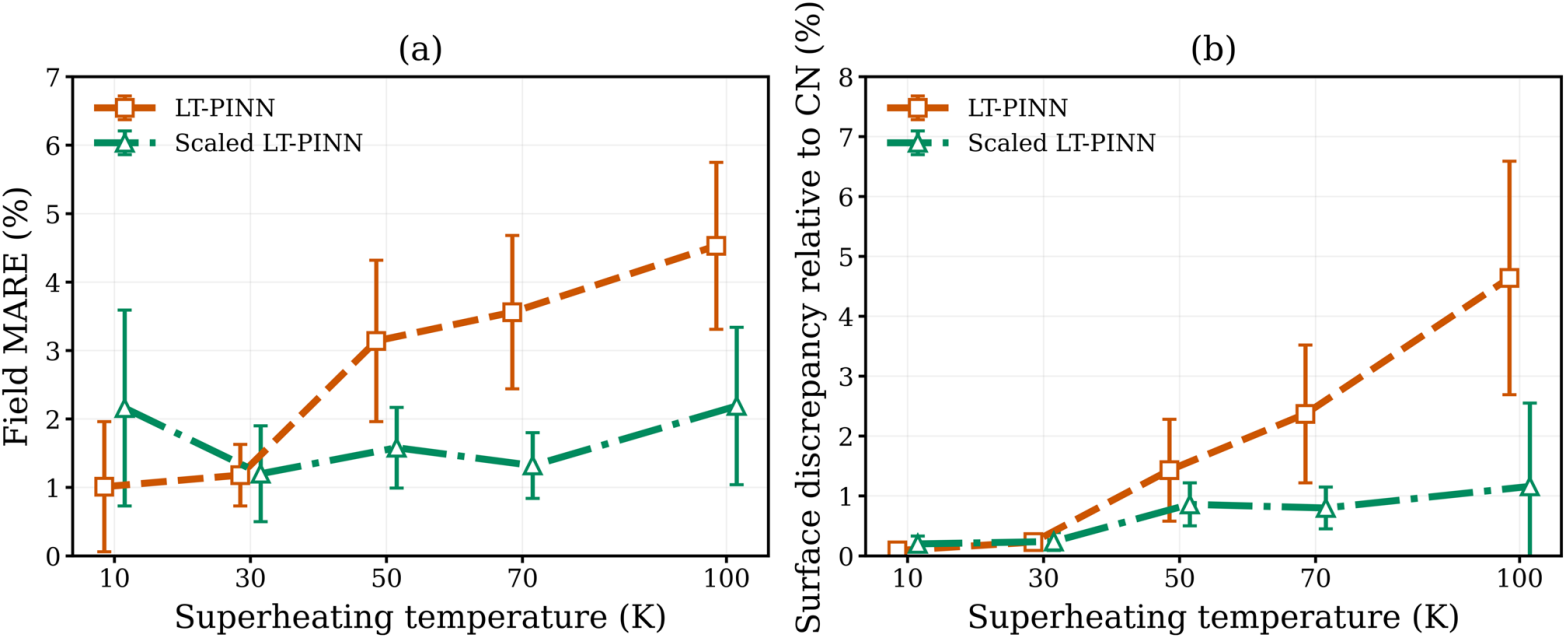
Error trends versus superheating temperature of LT-PINN and scaled LT-PINN (**a**) over the full spatial domain, (**b**) at the surface. Markers show mean values, and error bars denote standard deviations across five independent training seeds.

Figure 9 summarizes the temperature dependence of the LT-PINN and scaled LT-PINN discrepancies relative to the CN reference solution. Over the full domain, the LT-PINN exhibited an approximately monotonic increase in the field MARE with *ΔT*, reflecting the increasing stiffness and sharpening gradients. Because an increase in *ΔT* also corresponds to an increase in *Pe* in the present benchmark, this trend is consistent with a progressive increase in the dominance of *Pe*-driven convection. In contrast, the scaled LT-PINN maintained a comparatively flatter error response, indicating improved robustness across operating conditions rather than tuning to a narrow regime.

At the surface, the separation was stronger. The LT-PINN surface discrepancy increased rapidly at high *ΔT*, whereas the scaled LT-PINN increased more moderately and remained lower in the high-*ΔT* cases. This is consistent with the locking-time behavior. Because locking depends on the surface trajectory, the increasing surface error in LT-PINN helps explain the larger locking-time differences relative to the CN reference at *ΔT* = 70–100 K, whereas the scaled LT-PINN provides improved locking-time reliability in this high-*ΔT*, high-*Pe* regime. This trend is consistent with the intended role of the BC residual scaling, in which its effect remains limited when the *Pe*-driven boundary-loss imbalance is weak but becomes increasingly important in the high-*ΔT*, high-*Pe* regime, where boundary-layer steepening and stiffness amplify the training imbalance.

### Practical implications and limitations

The combined logarithmic transformation and *Pe*-based BC residual scaling provide a simple, physically interpretable strategy for the present class of stiff moving-boundary transport problems, in which a clear stiffness parameter exists and boundary-layer dynamics dominate the predictive utility. This method improves robustness without requiring additional machinery, such as adaptive sampling, decomposition, or specialized architectures. Accordingly, the present study should be interpreted as a controlled benchmark investigation of a one-dimensional, radially symmetric drying model rather than a complete validation of more realistic multiphysics or higher-dimensional superheated steam drying systems.

Several limitations should be noted. First, the scaling leverages problem knowledge (*Pe*); therefore, it presumes that the stiffness-controlling parameter is available and meaningful across regimes. Second, at moderate *ΔT*, scaling may not always yield the smallest error because it intentionally reduces the relative dominance of the BC residual in the loss function. This trade-off is justified by preventing high-*Pe* degradation and improving the reliability of the locking-time prediction in the stiff, high-*ΔT* regime. The remaining variability under extreme conditions suggests that additional complementary strategies may further reduce the error. However, the present approach substantially mitigates the dominant failure mechanism while preserving model simplicity.

A further limitation concerns the generalizability of the findings. The present formulation was derived and tested for a specific drying model with a clear stiffness structure: strong near-surface boundary layer formation, a wide dynamic range in the solution variable, and a physically interpretable stiffness parameter expressed in terms of *Pe*. Therefore, extending it to more realistic superheated steam drying models is not automatic. In higher-dimensional, multiphysics, or variable-property formulations, the most suitable transformed variable and residual scaling may differ, and the transformed equations must be rederived and revalidated on a case-by-case basis.

Another limitation is that the present validation is numerical rather than experimental. The proposed formulation was assessed against a numerically verified CN reference benchmark, which is appropriate for isolating formulation-level improvements but does not, by itself, establish predictive accuracy for real superheated steam drying experiments. Experimental validation against measured locking behavior, surface enrichment trends, droplet-size evolution, or related observables remains an important next step toward practical extensions.

## Future perspectives

Building on the findings, several future research directions can be identified.

i. Generalization across operating conditions and parameterized PINNs: The current study considers discrete levels of superheating. The natural next step is to train a single parameterized surrogate that takes *ΔT* (or *Pe*) as an additional input and predicts *Y(ξ, θ; ΔT)* over a continuum of conditions. This would enable rapid process optimization and uncertainty screening without retraining for each case and provide a more stringent test of whether the scaling strategy preserves accuracy under extrapolation in *Pe*. Once trained, the parameterized PINN could serve as a fast surrogate for the underlying drying model across a wider range of operating conditions.
ii. Multi-physics extensions beyond solute transport: Practical superheated steam drying and crust formation often involve additional coupled physics, including temperature gradients inside the droplet, evolving viscosity/porosity, and concentration-dependent diffusivity. Extending the present framework to variable-property models and coupled energy–mass transport would test the robustness of the transformation and scaling approach under stronger nonlinearities while maintaining the interpretability of the PINN formulation. However, such extensions would likely require the re-derivation of the transformed PDEs and reassessment of whether the same logarithmic transformation remains the most effective choice.
iii. Learning unknown closures and constitutive relations: Many drying models require closures (e.g., concentration-dependent diffusion, interfacial mass/heat transfer correlations, or effective permeability in a porous crust) that are uncertain and system-specific. A promising direction is to use the scaled LT-PINN as a hybrid inverse-modeling tool to infer such functions from limited measurements (e.g., droplet-radius history, surface-concentration proxies, or imaging-based crust thickness) while maintaining physical consistency.
iv. Experimental validation and process-level integration: Ultimately, the value of such surrogates is realized when they are integrated into process design loops. Because the present study was validated against a numerically verified reference model rather than direct experimental measurements, the next essential step is to validate it against experimental data on superheated steam drying. In particular, future work should compare the predicted locking times, surface enrichment trends, droplet-size evolution, and other experimentally accessible observables with measurements and embed the trained surrogates into optimization frameworks for energy use, product quality, and throughput.

## Conclusion

- Problem statement: This study addressed the persistent failure of the standard PINN formulation for stiff moving-boundary PDEs using the locking-point model for nanosuspension droplet drying in superheated steam as a challenging benchmark (*ΔT* = 10–100 K). Stiffness arises from rapid evaporation-driven convection and nonlinear solute enrichment, which produce sharp gradients near the droplet surface and render the accurate prediction of the locking event highly sensitive to surface fidelity.
- Proposed methodology: To overcome these instabilities, we investigated, for the present drying benchmark, a scaled logarithmically transformed PINN formulation (scaled LT-PINN) that combines two physics-guided modifications within the standard PINN backbone: (i) a logarithmic state transformation *q* = *lnY*, used here as a physics-guided reformulation to compress the solution dynamic range and mitigate training difficulty associated with the large variation in *Y*, and (ii) an inverse *Pe*-based surface BC residual scaling, implemented as a residual scaling of *1/(Pe* + *ε)*, to prevent the surface boundary term which grows approximately as *Pe*^*2*^ in the transformed loss, from overwhelming the PDE residual during training.
- Performance analysis: The ablation results confirmed the complementary roles of both formulation components. The baseline PINN failed under all operating conditions, showing an approximately constant field MARE of approximately 97% and failing to predict the locking time for any *ΔT*. The LT-PINN enabled physically meaningful solutions and accurate locking predictions under mild stiffness. However, its accuracy deteriorated systematically with increasing *ΔT*. In contrast, the scaled LT-PINN maintained more robust performance across the tested stiffness range, particularly in the high-*ΔT*, high-*Pe* regime. These improvements were accompanied by better resolution of the near-surface boundary layer and more reliable surface trajectories, which directly controlled the locking-time prediction.
- Generalizability: Overall, for the present one-dimensional, radially symmetric superheated steam drying benchmark, the scaled LT-PINN provides a simple, physically interpretable, and effective formulation-level strategy for stiff moving-boundary transport with steep near-boundary gradients and threshold-type events. These results should not be interpreted as proof of general transferability to arbitrary stiff PDEs. Rather, the present formulation may be useful for other convection-dominated moving-boundary systems in which the boundary terms introduce stiffness and loss imbalance. However, such extensions require case-specific derivation, testing, and validation.

## Electronic supplementary material

Below is the link to the electronic supplementary material.

Supplementary Information.

## Data Availability

The datasets used and/or analyzed during the current study are available from the corresponding author on reasonable request.

## Appendix A: Transformation from a moving to a fixed computational domain

In spherical coordinates, the diffusion equation is expressed as follows:

A.1 $$\frac{\partial {Y}_{i}}{\partial t}=D\hspace{0.17em}\frac{1}{{r}^{2}}\frac{\partial }{\partial r}\left({r}^{2}\frac{\partial {Y}_{i}}{\partial r}\right),\hspace{1em}0\le r\le a\left(t\right)$$

We map the physical radius *r* $\in \left[0,a\left(t\right)\right]$ to a fixed domain $\xi =\frac{r}{a\left(t\right)}\in \left[\mathrm{0,1}\right]$, so $r=a\left(t\right)\hspace{0.17em}\xi$. At a fixed *t,* $a\left(t\right)$ is constant, hence:

A.2 $${\left.\frac{\partial }{\partial r}\right|}_{t}=\frac{1}{a\left(t\right)}{\left.\frac{\partial }{\partial \xi }\right|}_{t}, {\left.\frac{{\partial }^{2}}{\partial {r}^{2}}\right|}_{t}=\frac{1}{a{\left(t\right)}^{2}}{\left.\frac{{\partial }^{2}}{\partial {\xi }^{2}}\right|}_{t}$$

For the spherical operator, we have

A.3 $${\left.\frac{1}{{r}^{2}}\frac{\partial }{\partial r}\left({r}^{2}\frac{\partial {Y}_{i}}{\partial r}\right)\right|}_{t}=\frac{1}{{a}^{2}{\xi }^{2}}{\left.\frac{\partial }{\partial \xi }\right|}_{t}\left({\xi }^{2}\frac{\partial {Y}_{i}}{\partial \xi }\right)$$

Because $r=\xi a\left(t\right)$, holding *r* fixed gives $dr = a\partial \xi + \hspace{0.17em}\xi da = 0$, so:

A.4 $${\left.\frac{\partial \xi }{\partial t}\right|}_{r}=-\frac{\xi }{a}\frac{da}{dt} =-\frac{\dot{a}}{a}\hspace{0.17em}\xi$$

Then:

A.5 $${\left.\frac{\partial {Y}_{i}}{\partial t}\right|}_{r}= {\left.\frac{\partial {Y}_{i}}{\partial t}\right|}_{\xi } +{\left.\frac{\partial {Y}_{i}}{\partial \xi }\right|}_{t}{\left.\frac{\partial \xi }{\partial t}\right|}_{r} = {\left.\frac{\partial {Y}_{i}}{\partial t}\right|}_{\xi }-\frac{\dot{a}}{a}\hspace{0.17em}\xi \hspace{0.17em}\frac{\partial {Y}_{i}}{\partial \xi }$$

For transforming Eq. (A.1), we use Eqs. (A.3) and (A.5); Eq. (A.1) becomes

A.6 $${\left.\frac{\partial {Y}_{i}}{\partial t}\right|}_{\xi }-\frac{\dot{a}}{a}\hspace{0.17em}\xi \hspace{0.17em}\frac{\partial {Y}_{i}}{\partial \xi }=\frac{D}{{a}^{2}{\xi }^{2}}\frac{\partial }{\partial \xi }\left({\xi }^{2}\frac{\partial {Y}_{i}}{\partial \xi }\right)$$

Multiplying by ${a}^{2}$ and using $a\dot{a}=\frac{1}{2}\frac{d\left({a}^{2}\right)}{dt}$ gives the fixed-domain equation:

A.7 $${a}^{2}\frac{\partial {Y}_{i}}{\partial t}=\frac{1}{2}\frac{d\left({a}^{2}\right)}{dt}\hspace{0.17em}\xi \hspace{0.17em}\frac{\partial {Y}_{i}}{\partial \xi }+\frac{D}{{\xi }^{2}}\frac{\partial }{\partial \xi }\left({\xi }^{2}\frac{\partial {Y}_{i}}{\partial \xi }\right)$$

## Appendix B: Time-scaling of the process model

To convert Eq. (19) to Eq. (22) using the definition of the new dimensionless time $\theta =-\mathrm{ln}\left(1+\alpha \tau \right)$, we used the chain rule between $\tau$ and $\theta$:

B.1 $$\theta =-\mathrm{ln}\left(1+\alpha \tau \right)$$

thus:

B.2 $$\frac{d\theta }{d\tau }=-\frac{\alpha }{1+\alpha \tau }$$

B.3 $$\frac{\partial }{\partial \tau }=\frac{d\theta }{d\tau }\frac{\partial }{\partial \theta } =-\frac{\alpha }{1+\alpha \tau }\hspace{0.17em}\frac{\partial }{\partial \theta }$$

B.4 $$\frac{\partial }{\partial \theta } = \frac{d\tau }{\partial \theta }\frac{\partial }{\partial \tau } = -\frac{1+\alpha \tau }{\alpha } \frac{\partial }{\partial \tau }$$

B.5 $$\frac{\partial {Y}_{i}}{\partial \theta } =\frac{d\tau }{\partial \theta }\frac{\partial {Y}_{i}}{\partial \tau } = -\frac{1+\alpha \tau }{\alpha } \frac{\partial {Y}_{i}}{\partial \tau }$$

Using Eq. (19), the transformed equation becomes:

B.6 $$\frac{\partial {Y}_{i}}{\partial \theta } =- \frac{G}{\alpha }\left[\frac{{\partial }^{2}{Y}_{i}}{\partial {\xi }^{2}}+\left(\frac{2}{\xi }-Pe\xi \right)\frac{\partial {Y}_{i}}{\partial \xi }\right]$$

## Appendix C: Verification of the CN reference solution

Because the main PINN results were evaluated against a CN reference solution, the CN discretization of the *Y*-space drying equation was verified prior to being used as a numerical benchmark. The reference discretization employed a conservative spatial formulation, second-order boundary elimination at *ξ* = *0* and *ξ* = *1*, and an implicit CN marching scheme in the transformed time variable, *θ*. The verification followed standard numerical practices and included checks for stability, consistency, convergence, and grid refinement of the actual problem.

First, the stability was assessed by analyzing the spectrum of the semi-discrete operator and the amplification behavior of the fully discrete CN scheme. In the verification runs, the largest amplification factor over the examined dissipative modes was 0.999958, which is slightly below unity. This provides evidence of modal stability for the tested discretizations, because the CN amplification factors associated with the examined dissipative modes remained bounded. The stability assessment was therefore based on both the CN amplification behavior and the observed boundedness of the refined numerical solutions.

Second, consistency was examined using a manufactured solution local truncation-residual study. The discrete CN residual was evaluated directly using the manufactured exact solution and corresponding analytic source term on successively refined grids. The residual decreased systematically during refinement, and the median observed order, based on the mean local truncation error, was 2.476, which supported the expected second-order truncation behavior of the CN discretization for the tested manufactured solution. Third, convergence was assessed using an MMS-based coupled space–time refinement study. The modified governing equation with the manufactured source term was solved on successively refined spatial and temporal grids, and the observed order was computed from the resulting discretization error. The median observed convergence order was 2.167, indicating convergence consistent with the expected second-order behavior of the CN discretization for the verified *Y*-space formulation.

Finally, an actual-problem grid refinement study was performed on the transformed drying problem. For this study, four coupled space–time discretizations were used: (*Nx, N*_*θ*_) = (201,800), (401,1600), (801,3200), and (1601,6400). The finest grid (1601,6400) was used as the reference level, and the coarser solutions were compared against it on a common evaluation grid of 200 spatial points and 60 transformed-time levels. Because no exact analytical solution is available for the actual drying problem, this study was used as a grid sensitivity assessment rather than a formal exact-error estimate. For all the investigated superheating temperatures, the mean relative L_2_​ difference between the coarse and refined CN solutions decreased significantly with refinement. Specifically, the mean relative difference decreased from 1.440 × 10^−3^ to 7.679 × 10^−5^ at 10 K, from 1.670 × 10^−2^ to 8.251 × 10^−4^ at 30 K, from 6.399 × 10^−2^ to 3.017 × 10^−3^ at 50 K, from 1.764 × 10^−1^ to 7.401 × 10^−3^ at 70 K, and from 7.846 × 10^−1^ to 1.986 × 10^−2^ at 100 K. This indicates grid sensitivity decreased under refinement for every superheating temperature considered. The 100 K case remained the most demanding, as expected from the stronger near-surface gradients in this regime. Taken together, these results support the use of the CN solution as a numerically verified reference benchmark for the PINN comparisons reported in the main text. In the final reevaluation reported in this study, the saved PINN models were compared with the verified *Y*-space CN reference.

## Appendix D: Additional robustness study of the baseline PINN

This appendix summarizes the additional robustness study performed on the baseline PINN. This study tested whether stronger tuning of the baseline alone could substantially improve its performance beyond the common settings used in the main comparison.

The study was conducted in staged sweeps. First, the baseline architecture was varied using combinations of network width and depth. Second, optimizer-related settings were explored, including the learning rate, scheduler use, and whether early stopping was enabled or not. Third, the collocation sensitivity was examined by varying the number of PDE and surface collocation points. Finally, the strongest candidate from these preliminary sweeps was re-evaluated using five random seeds. Unless otherwise stated, the robustness runs used the same Adam budget (up to 10,000 epochs) and L-BFGS refinement as the main study. Table 4 summarizes the tested settings and best observed outcomes for each stage.﻿﻿

**Table 4:** Summary of additional robustness tests for the baseline PINN.

Stage	Tested settings	Best observed result	Conclusion
Architecture sweep	Width = 64, 128, 256; Depth = 3, 5, 7	Field MARE = 96.75%; no locking prediction	Architecture changes alone did not rescue the baseline
Optimizer sweep	Learning rate = 10^–4^, 5 × 10^–4^, 10^–3^; scheduler on/off; early stopping on/off	Field MARE = 90.80 ± 8.60%; no locking prediction	Stronger optimizer tuning still did not resolve baseline failure
Collocation sweep	${N}_{PDE}=\mathrm{1,000}, \mathrm{2,000}, \mathrm{4,000}, {N}_{surface}=500, \mathrm{1,000}, \mathrm{2,000}$	Field MARE = 81.93 ± 15.04%; no locking prediction	Increased collocation density improved the error only marginally and did not restore locking prediction
Final 5-seed evaluation	Best finalist tested across 5 seeds	Field MARE = 90.31 ± 11.39%; no locking prediction	Even the strongest tuned baseline remained qualitatively unreliable

The results summarized in Table 4 support the interpretation in the main text that the failure of the baseline PINN is not merely due to insufficient tuning but is rooted in the difficulty of learning the original stiff formulation.

## List of symbols

A: Droplet surface area (m^2^)
a(t): Time-dependent droplet radius (m)
$\overline{a }$: Dimensionless droplet radius (–)
D: Binary diffusion coefficient of nanoparticles in the liquid (m^2^ s^−1^)
d: Droplet diameter (m)
G: Dimensionless diffusion group (–)
K: Number of evaluation times used to compute field MARE (–)
k_B_: Boltzmann constant (J K^−1^)
${\dot{m}}_{i}$: Mass transfer rate of species *i* across the droplet surface (kg s^−1^)
N_PDE_: Number of PDE collocation points (–)
N_surface_: Number of surface boundary collocation points (–)
N_x_: Number of spatial evaluation points (–)
Nu: Nusselt number (–)
p: Index over trainable network parameters (–)
Pe: Péclet number (–)
q: Logarithmically transformed concentration variable (–)
r: Physical radial coordinate (m)
r_p_: Nanoparticle radius (m)
t: Dimensional time (s)
t_l_: Droplet lifetime used for nondimensionalization (s)
T_d_: The boiling temperature of the liquid (K)
w_p_: Trainable network parameter (–)
Y: Solute mass fraction (–)
α: Dimensionless shrinkage parameter (–)
Δh_v_: Specific evaporation enthalpy (J kg^−1^)
ΔT: Superheating temperature (K)
δ: Early-stopping threshold (–)
ε: Small positive constant used for numerical stability and boundary condition residual scaling (–)
η_l_: Dynamic viscosity of the nanosuspension (Pa s)
θ: Log-time transformed dimensionless time (–)
κ: Evaporation/shrinkage constant in the d^2^-law (m^2^ s^−1^)
λ_vap_: Thermal conductivity of the superheated vapor (W m^−1^ K^−1^)
ρ: Mass density (kg m^−3^)
τ: Dimensionless drying time (–)
ξ: Fixed dimensionless radial coordinate (–)
ω: Weight assigned to each loss term (–)

## Subscripts/superscripts

0: Initial value
*i*: Loss-component or species index, depending on context
*l*: Liquid phase
*s*: Solid phase or surface value, depending on context

## Abbreviations

BC: Boundary condition
CN: Crank–Nicolson
Field MARE: Field mean absolute relative error
IC: Initial condition
L-BFGS: Limited-memory Broyden–Fletcher–Goldfarb–Shanno optimizer
LT-PINN: Logarithmically transformed physics-informed neural network
MLP: Multilayer perceptron
MMS: Method of manufactured solutions
MSE: Mean squared error
NTK: Neural tangent kernel
PDE: Partial differential equation
PINN: Physics-informed neural network
Scaled LT-PINN: Logarithmically transformed PINN with Péclet-number-based BC residual scaling

## References

[1] Samborska, K. et al. Recent progress in the stickiness reduction of sugar-rich foods during spray drying. *Dry. Technol.* **41**, 2566–2585 (2023).

[2] Bück, A. Model for structure formation in nanosuspension droplet superheated steam drying. *Dry. Technol.* **42**, 2070–2079 (2024).

[3] Sobulska, M., Wawrzyniak, P. & Woo, M. W. Superheated steam spray drying as an energy-saving drying technique: A review. *Energies* **15**, 8546. 10.3390/en15228546 (2022).

[4] Karniadakis, G. E. et al. Physics-informed machine learning. *Nat. Rev. Phys.* **3**, 422–440. 10.1038/s42254-021-00314-5 (2021).

[5] Haghighat, E., Raissi, M., Moure, A., Gomez, H. & Juanes, R. A physics-informed deep learning framework for inversion and surrogate modeling in solid mechanics. *Comput. Methods Appl. Mech. Eng.* **379**, 113741. 10.1016/j.cma.2021.113741 (2021).

[6] Malekjani, N., Kharaghani, A. & Tsotsas, E. A comparative study of dimensional and non-dimensional inputs in physics-informed and data-driven neural networks for single-droplet evaporation. *Chem. Eng. Sci.* **306**, 121214. 10.1016/j.ces.2025.121214 (2025).

[7] Malekjani, N., Kharaghani, A. & Tsotsas, E. Physics-informed and data-driven neural networks with dimensional and non-dimensional inputs for single-droplet evaporation: investigating the role of increasing physical complexity in predictive ability. *Chem. Eng. Sci.* **316**, 121911. 10.1016/j.ces.2025.121911 (2025).

[8] Malekjani, N., Kharaghani, A. & Tsotsas, E. Physics-informed vs. data-driven neural networks for n-heptane droplet evaporation. *Chem. Eng. Res. Des.* **228**, 469–483 (2026).

[9] Daw, A., Bu, J., Wang, S., Perdikaris, P. & Karpatne, A. Mitigating propagation failures in physics-informed neural networks using retain-resample-release (R3) sampling. In *Proceedings of the 40th International Conference on Machine Learning* **202,** 7264–7302 (PMLR, 2023).

[10] Krishnapriyan, A. S., Gholami, A., Zhe, S., Kirby, R. M. & Mahoney, M. W. Characterizing possible failure modes in physics-informed neural networks. *Adv. Neural Inf. Process. Syst.* **34**, 26548–26560 (2021).

[11] Wang, S., Wang, H. & Perdikaris, P. On the eigenvector bias of Fourier feature networks: from regression to solving multi-scale PDEs with physics-informed neural networks. *Comput. Methods Appl. Mech. Eng.* **384**, 113938. 10.1016/j.cma.2021.113938 (2021).

[12] Malekjani, N., Bück, A., Kharaghani, A. & Tsotsas, E. SDD-PINN: physics-informed neural network for single droplet drying. *Digit. Chem. Eng.* **19**, 100306. 10.1016/j.dche.2026.100306 (2026).

[13] Rahaman, N. et al. On the spectral bias of neural networks. *In Proceedings of the 36th International Conference on Machine Learning*, **97,** 5301–5310 (PMLR, 2019).

[14] Wang, S., Yu, X. & Perdikaris, P. When and why PINNs fail to train: a neural tangent kernel perspective. *J. Comput. Phys.* **449**, 110768. 10.1016/j.jcp.2021.110768 (2022).

[15] Wu, C., Zhu, M., Tan, Q., Kartha, Y. & Lu, L. A comprehensive study of non-adaptive and residual-based adaptive sampling for physics-informed neural networks. *Comput. Methods Appl. Mech. Eng.* **403**, 115671. 10.1016/j.cma.2022.115671 (2023).

[16] Guo, J., Wang, H., Gu, S. & Hou, C. TCAS-PINN: physics-informed neural networks with a novel temporal causality-based adaptive sampling method. *Chin. Phys. B* **33**, 050701. 10.1088/1674-1056/ad21f3 (2024).

[17] Moseley, B., Markham, A. & Nissen-Meyer, T. Finite basis physics-informed neural networks (FBPINNs): A scalable domain decomposition approach for solving differential equations. *Adv. Comput. Math.* **49**, 62. 10.1007/s10444-023-10065-9 (2023).

[18] Cao, F., Gao, F., Guo, X. & Yuan, D. Physics-informed neural networks with parameter asymptotic strategy for learning singularly perturbed convection-dominated problem. *Comput. Math. Appl.* **150**, 229–242 (2023).

[19] Boro, P., Raina, A. & Natesan, S. A parameter-driven physics-informed neural network framework for solving two-parameter singular perturbation problems involving boundary layers. *Adv. Comput. Sci. Eng.* **5**, 72–102. 10.3934/acse.2025019 (2025).

[20] Wang, S., Teng, Y. & Perdikaris, P. Understanding and mitigating gradient flow pathologies in physics-informed neural networks. *SIAM J. Sci. Comput.* **43**, A3055–A3081 (2021).

[21] Jagtap, A. D. & Karniadakis, G. E. Extended physics-informed neural networks (XPINNs): a generalized space-time domain decomposition based deep learning framework for nonlinear partial differential equations. *Commun. Comput. Phys.* **28**, 2002–2041 (2020).

[22] Wang, S., Wang, H. & Perdikaris, P. Learning the solution operator of parametric partial differential equations with physics-informed DeepONets. *Sci. Adv.* **7**, eabi8605. 10.1126/sciadv.abi8605 (2021). 34586842 10.1126/sciadv.abi8605 PMC8480920

[23] Guan, J. & Elman, H. Transformed physics-informed neural networks for the convection-diffusion equation. Preprint at https://arxiv.org/abs/2409.07671 (2024).

[24] Amiri, A., Barrenechea, G. R. & Pryer, T. A nodally bound-preserving finite element method for time-dependent convection–diffusion equations. *J. Comput. Appl. Math.* **470**, 116691. 10.1016/j.cam.2025.116691 (2025).

[25] Priyanka, Sahani, S., Mebarek-Oudina, F. & Arora, S. Computationally efficient numerical technique to study 2D time fractional Burger’s equation. *Arab. J. Math.*10.1007/s40065-026-00635-2 (2026).

[26] Priyanka, Arora, S., Mebarek-Oudina, F. & Sahani, S. Super convergence analysis of fully discrete Hermite splines to simulate wave behavior of Kuramoto-Sivashinsky equation. *Wave Motion* **121**, 103187. 10.1016/j.wavemoti.2023.103187 (2023).

[27] Priyanka, Mebarek-Oudina, F., Sahani, S. & Arora, S. Travelling wave solution of fourth order reaction diffusion equation using hybrid quintic Hermite splines collocation technique. *Arab. J. Math.* **13**, 341–367 (2024).

[28] Farhan, M. et al. Implementation of the one-step one-hybrid block method on the nonlinear equation of a circular sector oscillator. *Comput. Math. Model.* **31**, 116–132 (2020).

[29] Chaurasiya, V. Numerical simulation of a non-linear sublimation process with temperature-dependent permeability and volumetric heat source: a phase change problem. *Comput. Math. Appl.* **176**, 55–66 (2024).

[30] Chaurasiya, V., Rajeev & Kumar, J. Bernstein operational matrix of differentiation to analyze the conductive, convective, and radiative non-linear sublimation model with temperature-dependent internal heat generation. *J. Therm. Stresses* **47**, 1450–1478 (2024).

[31] Chaurasiya, V., Shah, N. A., Kumar, P. & Sharma, S. K. Gegenbauer wavelet approach of a two-phase moving boundary problem with functionally graded phase change material. *Therm. Sci. Eng. Prog.* **68**, 104196. 10.1016/j.tsep.2025.104196 (2025).

[32] Shah, N. A., Kumar, N., Yadav, H. C. & Chaurasiya, V. Gegenbauer wavelet collocation method to analyze one-dimensional solid–liquid phase change in a functionally graded material with liquid fraction distribution. *Int. Commun. Heat Mass Transf.* **162**, 108620. 10.1016/j.icheatmasstransfer.2025.108620 (2025).

[33] Brenn, G. Concentration fields in drying droplets. *Chem. Eng. Technol.* **27**, 1252–1258 (2004).

[34] Xu, Z.-Q.J., Zhang, L. & Cai, W. On understanding and overcoming spectral biases of deep neural network learning methods for solving PDEs. *J. Comput. Phys.* **530**, 113905. 10.1016/j.jcp.2025.113905 (2025).

[35] Goswami, A. & Patel, K. S. Matrix method stability and robustness of compact schemes for parabolic PDEs. Preprint at https://arxiv.org/abs/2201.05854 (2022).

[36] Thomas, J. L., Diskin, B. & Rumsey, C. L. Towards verification of unstructured-grid solvers. *AIAA J.* **46**, 3070–3079 (2008).

[37] Veluri, S. P., Roy, C. J. & Luke, E. A. Comprehensive code verification techniques for finite volume CFD codes. *Comput. Fluids* **70**, 59–72 (2012).

[38] Roy, C. J. Grid convergence error analysis for mixed-order numerical schemes. *AIAA J.* **41**, 595–604 (2003).

[39] Lu, L. et al. Physics-informed neural networks with hard constraints for inverse design. *SIAM J. Sci. Comput.* **43**, B1105–B1132 (2021).

