# Supplementary material for: PINN for stiff moving-boundary PDE to predict the locking point in superheated steam drying
Source: Sci Rep. 2026 Jun 23;16:19548. doi: 10.1038/s41598-026-59320-1 (PMC13291356; doi:10.1038/s41598-026-59320-1)
Supplement: Supplementary file 1 — Supplementary Information. [file 41598_2026_59320_MOESM1_ESM.docx]

# Supplementary Material

This supplementary figure shows the total-loss convergence histories for the baseline PINN, LT-PINN, and scaled LT-PINN at the investigated superheating temperatures.

.
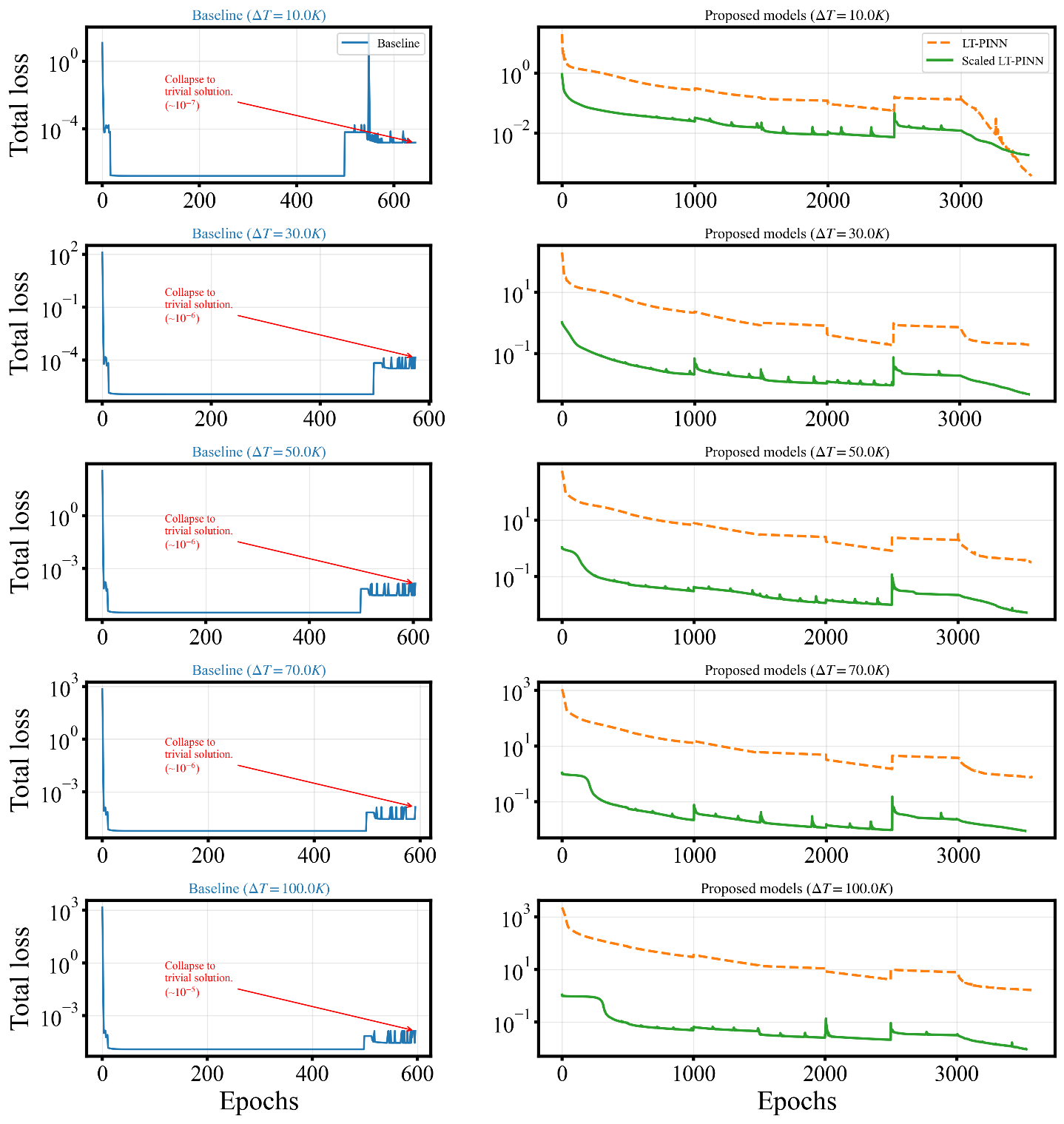


Fig. S1. Total training-loss convergence histories of the baseline PINN, LT-PINN, and scaled LT-PINN across superheating temperatures.
